# Modeling Transition Metals in East Asia and Japan and Its Emission Sources

**DOI:** 10.1029/2020GH000259

**Published:** 2020-09-14

**Authors:** Mizuo Kajino, Hiroyuki Hagino, Yuji Fujitani, Tazuko Morikawa, Tetsuo Fukui, Kazunari Onishi, Tomoaki Okuda, Tomoki Kajikawa, Yasuhito Igarashi

**Affiliations:** ^1^ Meteorological Research Institute (MRI), Japan Meteorological Agency (JMA) Tsukuba Japan; ^2^ Faculty of Life and Environmental Sciences University of Tsukuba Tsukuba Japan; ^3^ Japan Automobile Research Institute (JARI) Tsukuba Japan; ^4^ National Institute for Environmental Studies (NIES) Tsukuba Japan; ^5^ Institute of Behavioral Sciences Tokyo Japan; ^6^ Graduate School of Public Health St. Luke's International University Tokyo Japan; ^7^ Faculty of Science and Technology Keio University Yokohama Japan; ^8^ Graduate School of Creative Science and Engineering Waseda University Tokyo Japan; ^9^ Institute for Integrated Radiation and Nuclear Science (KURNS) Kyoto University Osaka Japan; ^10^ College of Science Ibaraki University Mito Japan

**Keywords:** East Asia, emission inventory, Japan, numerical modeling, oxidative potential, transition metals

## Abstract

Emission inventories of anthropogenic transition metals, which contribute to aerosol oxidative potential (OP), in Asia (Δ*x* = 0.25°, monthly, 2000–2008) and Japan (Δ*x* = 2 km, hourly, mainly 2012) were developed, based on bottom‐up inventories of particulate matters and metal profiles in a speciation database for particulate matters. The new inventories are named Transition Metal Inventory (TMI)‐Asia v1.0 and TMI‐Japan v1.0, respectively. It includes 10 transition metals in PM_2.5_ and PM_10_, which contributed to OP based on reagent experiments, namely, Cu, Mn, Co, V, Ni, Pb, Fe, Zn, Cd, and Cr. The contributions of sectors in the transition metals emission in Japan were also investigated. Road brakes and iron‐steel industry are primary sources, followed by other metal industry, navigation, incineration, power plants, and railway. In order to validate the emission inventory, eight elements such as Cu, Mn, V, Ni, Pb, Fe, Zn, and Cr in anthropogenic dust and those in mineral dust were simulated over East Asia and Japan with Δ*x* = 30 km and Δ*x* = 5 km domains, respectively, and compared against the nation‐wide seasonal observations of PM_2.5_ elements in Japan and the long‐term continuous observations of total suspended particles (TSPs) at Yonago, Japan in 2013. Most of the simulated elements generally agreed with the observations, while Cu and Pb were significantly overestimated. This is the first comprehensive study on the development and evaluation of emission inventory of OP active elements, but further improvement is needed.

## Key Points

- Emission inventories of transition metals, which contribute to aerosol oxidative potential (OP), in Asia and Japan were developed
- Most of simulated elements (Mn, V, Ni, Fe, Zn, and Cr) agreed with observation, while Cu and Pb were significantly overestimated
- Road brakes and iron‐steel industry are primary sources, followed by metal industry, navigation, incineration, power plants, and railway

## 1 Introduction

The dry mass of aerosols smaller than 2.5 μm in diameter (50% cutoff ambient aerodynamic diameter is 2.5 μm), defined as PM_2.5_, is strongly associated with adverse health effects (Pope et al., 2002; Pope & Dockery, 2006; Lelieveld et al., 2015). However, its chemical composition varies in time and space; thus, it is assumed that the toxicity of PM_2.5_ and its health impacts also vary in time and space. There should be a highly toxic PM_2.5_ and a less toxic PM_2.5_. For example, one of the major chemical compounds of PM_2.5_, ammonium sulfate, has negligible toxicity (Grahame & Schlesinger, 2005). On the other hand, PM_2.5_ consists of several different chemical compounds, and their toxicities vary substantially depending on the compounds. The contributions of each compound to adverse health effects are impossible to determine. This complexity may explain why epidemiological studies have proven that PM_2.5_, with a mixture of varieties, is a good proxy for the adverse health effects of aerosols. In addition, PM_2.5_ is more easily measured than each chemical compound. Thus, the environmental standard was set for the surface air concentration of PM_2.5_, and the concentrations have been extensively monitored in various countries. The air quality guideline for the 24‐hr concentration is 25 μg m^−3^ (World Health Organization [WHO], 2005). The air quality standard depends on the standard set by each country, but 35 μg m^−3^ is the standard that has been selected in the United States, Japan, and many countries. Using PM_2.5_ as a proxy may be safe for monitoring the health hazards of aerosols, but it could mislead the development of emission reduction strategies. It is nonsensical to reduce less toxic compounds, such as ammonium sulfate, only to reduce mass concentrations.

Recently, the oxidative potential (OP) of aerosols (the potential to generate reactive oxygen species [ROS] in cells and cause oxidative stress to cells) has been focused on as a health hazard of aerosols (Jiang et al., 2019; Shiraiwa et al., 2017). A model calculation of ROS production in the epithelial lining fluid (ELF) using PM_2.5_ measurements from various locations around the world showed that ROS production varied by threefold to fourfold for the same PM_2.5_ concentration (e.g., the simulated ROS concentration varied from 50 to 200 nmol L^−1^ for 25–35 μg m^−3^ for the PM_2.5_ mass) (Lakey et al., 2016). They showed that 100 nmol L^−1^ as the ROS level characteristic of healthy humans. Even for the PM_2.5_ levels close to the air quality standard (25–35 μg m^−3^), in terms of ROS production, there may be highly toxic PM_2.5_ (producing 200 nmol L^−1^ of ROS) and less toxic PM_2.5_ (producing 50 nmol L^−1^ of ROS). Several assays have been established to quantify the OP of aerosols (Hedayat et al., 2015). The dithiothreitol ((2*S*,3*S*)‐1,4‐bis (sulfanyl)butane‐2,3‐diol; DTT) assay, developed by Kumagai et al. (2002), is one of the widely used ways to mimic the *in vivo* generation of superoxide radicals. OP is quantified as the consumption rate of the reducing agent, i.e., DTT in Tris‐HCl buffer, where aerosols are extracted. The DTT activity correlates well with heme oxygenase‐1 induction, which is an indicator of oxidative stress in living organisms (Li et al., 2003). DTT activity was found to be more strongly associated with emergency department (ED) visits related to asthma/wheezing and congestive heart failure than PM_2.5_ (Bates et al., 2015). The population‐level analysis of the health effect of measured ambient DTT reported that the 3‐D moving average of DTT activity was strongly associated with ED visits, especially for ischemic heart disease (Abrams et al., 2017). Charrier and Anastasio (2012) reported that the observed DTT was successfully explained by 80% of water‐soluble transition metals, such as Cu (II), Mn (II), Fe (II), and Fe (III), and 20% of organics, such as phenanthrenequinone (Charrier & Anastasio, 2012). On the other hand, Nishita‐Hara et al. (2019) reported that water‐soluble transition metals could explain only 37% and 60% of the measured DTT activity of fine and coarse particles, respectively. Saffari et al. (2014a) reported that DTT activity was strongly associated with water‐soluble and water‐insoluble organics, hopanes, and even elemental carbon. In fact, dissolved oxygen caused interfacial catalytic oxidation of DTT in the presence of elemental carbon particles (Sauvain & Rossi, 2016). Verma et al. (2015) reported the importance of humic‐like substances (HULIS) such as quinones and secondary organic aerosols in ambient OP, and Yu et al. (2018) reported interactions of HULIS, and transition metals should be considered for the DTT activity. In addition to the catalytic redox reactions of transition metals and quinones, noncatalytic DTT active organics such as organic hydro peroxides and electron‐deficient alkenes have been recently highlighted (Jiang et al., 2020). Thus far, the relative importance of chemical compositions to OP has not been well understood, but the importance of coexistence of metals and organics is a consensus (e.g., Charrier & Anastasio, 2012; Fang et al., 2019; Jiang et al., 2019; Saffari et al., 2014b; Shiraiwa et al., 2017; Yu et al., 2018).

While a large number of three‐dimensional modeling studies have been conducted for PM_2.5_, none have been performed for OP. Toward the simulation of aerosol OP as a final goal, as a first step, we developed emission inventories of OP active elements in Asia and Japan and developed a three‐dimensional transport model to evaluate the emission inventories by using the surface air concentrations measurements in Japan. There have been several studies for the development and evaluation of emission inventories of metals (Table 1). Reff et al. (2009) developed an anthropogenic emission inventory of dozens of hazardous trace elements in PM_2.5_ in the United States, which was evaluated by transport models and observation data across the United States and Canada (Appel et al., 2013; Xu et al., 2019). Dore et al. (2014) evaluated the toxic heavy metals in total suspended particle (TSP) emission in the United Kingdom with a transport model. Nickel et al. (2017) simulated the heavy metal concentrations in moss across Europe, as they are toxic to plants (Nagajyoti et al., 2010). Tian et al. (2015) developed an anthropogenic emission inventory of toxic heavy metals of TSPs in China, which was evaluated by a transport model and observation data in a highly polluted region of Northern China (Liu et al., 2019). Ying et al. (2018) developed both anthropogenic and natural (mineral dust [MD]) inventories of hazardous trace elements in China and evaluated by a transport model and observation data in mega cities in China such as Beijing, Nanjing, and Chengdu. The main objectives of the previous studies are toxicity of heavy metals (Bhanarkar et al., 2005; Gargava et al., 2014; Reff et al., 2009; Tian et al., 2015; Ying et al., 2018), aqueous phase catalytic oxidation (e.g., Fe [III] and Mn [II]) (Fu et al., 2016; Itahashi, Yamaji, Chatani, & Hayami, 2018; Itahashi, Yamaji, Chatani, Hisatsune, et al., 2018), marine primary productivity (Fe) (Xuan, 2005; Zhang et al., 2015), and light‐absorbing climate forcer (iron oxides) (Ito et al., 2018; Matsui et al., 2018). Thus far, no studies have been focusing on OP, and thus, the selections of elements are sometimes in common but basically different from the current study (Table 1): Cu was missing in the catalytic oxidation studies, while Fe was missing in the toxicity studies. Among the toxicity studies, the previous studies focused on PM_2.5_, while we also considered PM_10_. PM_2.5_ is believed to deposit deeper in the lung than coarse mode particles; however, lung deposition of PM_10_ may not be negligible because the deposition ratio of course mode particles is enhanced for some human condition cases (e.g., oral inhalation) (RIVM, 2002). Asian MD particles, which contain metals and exist mostly in the coarse mode, can have adverse effects on health (Hashizume et al., 2010). The PM_2.5_ to PM_10_ ratio of metals, which we estimated, is important because of its water‐soluble DTT activity, because some of transition metals such as Cu, Mn, and Fe become more water‐soluble as aerosol acidity is higher, and aerosol acidity is associated with its size; e.g., aerosol pH is lower in finer particles as they contain more acids such as sulfate and nitrate (Fang et al., 2017).

**Table 1:** List of Studies on Emission Inventories and Transport Models for Metals

Reference	Methodology	Aerosol type	Region	DTT active elements^a^
Bhanarkar et al. (2005)	EI	PM^b^	Mumbai	Cu, Mn, V, and Ni
Xuan (2005)	EI^c^	MD^d^	E. Asia	Mn and Fe
Reff et al. (2009)	EI	PM_2.5_ ^e^	US	Mn and Ni
Appel et al. (2013)	TM^f^	PM_2.5_	US	Mg and Fe
Dore et al. (2014)	TM	PM	UK	Cu, Ni, and V
Gargava et al. (2014)	EI	PM_10_ ^g^	Delhi	Ni and V
Tian et al. (2015)	EI	PM	China	Cu, Mn, and Ni
Zhang et al. (2015)	EI and TM	MD	Global	Mn and Fe
Fu et al. (2016)	EI and TM	PM_2.5_ and MD	China	Mn (II) and Fe (III)
Nickel et al. (2017)	TM	PM	Europe	Cu, V, and Ni
Itahashi, Yamaji, Chatani, and Hayami (2018) and Itahashi, Yamaji, Chatani, Hisatsune, et al. (2018)	EI and TM	PM_2.5_ and PM_10_	Japan	Mn and Fe
Ying et al. (2018)	EI and TM	PM_2.5_ and MD	China	Cu, Mn, and Fe
Liu et al. (2019)	TM	PM_2.5_	N. China	Cu, Mn, and Ni
Xu et al. (2019)	TM	PM_2.5_ and MD	N. America	Fe, Mn, and Ni
This study	EI and TM	PM_2.5_ and PM_10_, MD	Asia and Japan	Cu, Mn, Fe, V, and Ni

^a^ Top five DTT active elements available in the literatures. Note that there are other elements studied in the literatures.

^b^ Anthropogenic particulate matter (no size information).

^c^ Emission inventory (EI) development.

^d^ Mineral dust (MD).

^e^ Anthropogenic PM_2.5_.

^f^ Evaluation of EI using transport models (TMs) and field measurements.

^g^ Anthropogenic PM_10_.

The development of emission inventories and the description of the numerical model are presented in section 2. The field observation data in Japan described in section 3 were used for the evaluation of the emission inventories and the numerical model. The results and discussion are presented in section 4, and concluding remarks are summarized in section 5. Ten transition metals, which consumed DTT as presented by Charrier and Anastasio (2012), were selected: Cu, Mn, Co, V, Ni, Pb, Fe, Zn, Cd, and Cr. We showed the top five DTT consumers in the air (i.e., nmol‐DTT min^−1^ m^−3^‐air), as provided by two previous studies using reagent experiments and field measurements in the United States (Charrier & Anastasio, 2012) and in Japan (Fujitani et al., 2017), namely, Cu, Mn, Fe, V, and Ni, in the main text and the others in Appendix A. However, we did not publish the results of Co and Cd because the emissions of these elements have not been evaluated by the observations. There were no observation data available for Co and Cd because their surface air concentrations in Japan were lower than their detection limits. Thus, we presented only the results of Pb, Zn, and Cr in Appendix A. However, we presented the emission factors of all 10 elements in the Supporting Information S1.

## 2 Methods

### 2.1 Semi‐Bottom‐Up Approach for Emission Inventory of Transition Metals

The bottom‐up emission inventory was compiled by the following equation (e.g., Kurokawa et al., 2013):

(1) $$E_{B,k}=\sum_{i,j}A_{i,j}\times {\mathrm{EF}}_{i,j}\times \left(1-R_{i,j}\right),$$

where *E*_*B,k*_ is the bottom‐up emission flux of sector *k* (published and thus available) from each country and subregion and *i* and *j* are the fuel and subsector types, respectively. *A*_*i,j*_, *EF*_*i,j*_, and *R*_*i,j*_ are the fuel consumption rate (or mileage for mobile sources), unabated emission factors, and removal efficiency from abatement technology, respectively. Unique values are provided for each variable, and expert judgment was made for the uncertainty estimation (e.g., Kurokawa et al., 2013; Streets et al., 2003). Among the metal inventories listed in Table 1, Bhanarkar et al. (2005) and Tian et al. (2015) provided the bottom‐up emission inventories.

On the other hand, in this study, to obtain the emission flux of transition metals, we used the following technique by using the existing bottom‐up emission inventories of PM_2.5_ and PM_10_ (hereafter referred to as PM_x_) and the United States Environmental Protection Agency (US EPA, 2014) SPECIATE database v4.4. The database is available at https://www.epa.gov/air-emissions-modeling/speciate (last accessed: 15 April 2019). Tens of sectors are usually provided by the bottom‐up inventories, while 3,217 metal profiles in PM_x_ are provided by SPECIATE v4.4. Consequently, the metal profiles were grouped corresponding to each sector of the bottom‐up inventories and multiplied by the bottom‐up PM_x_ emission fluxes. We named this method the semi‐bottom‐up approach. The same approach was taken in the previous metal inventories such as Gargava et al. (2014), Reff et al. (2009), Xuan (2005), and Ying et al. (2018) in Table 1. The previous bottom‐up and semi‐bottom‐up inventories provided unique values of emission fluxes by using unique emission factors or unique metal profiles for each sector. In this study, in order to estimate the uncertainty or variations in the metal profiles for each sector, the maximum, average, and minimum values of profiles were multiplied by the bottom‐up emission fluxes of PM_x_, as expressed by the following equation:

(2) $$E_{\mathrm{SB}}\left\{\begin{array}{c} \max\\ \mathrm{ave}\\ \min \end{array}\right\}=\sum_{k}E_{B,k}\times P_{k}\left\{\begin{array}{c} \max\\ \mathrm{ave}\\ \min \end{array}\right\},$$

where *E*_SB_ is the semi‐bottom‐up emission flux (kg PM y^−1^) and *P*_*k*_ is the metal profile in PM_x_ (kg metal kg PM^−1^). Note that this equation does not provide unique emission fluxes for each element. Instead of providing uncertainty, it provides the maximum, average, and minimum estimates of their emission fluxes. The *E*_*B,k*_ values are unique with some uncertainties coming from the right hand terms in Equation 1. Because SPECIATE does not provide metal profiles of railway emissions, the Tokyo Metropolitan Government's (TMG, 2011a) profile was used. Thus, the railway emission had unique values.

Table 2 summarizes the bottom‐up emission inventories used in this study. For Asia, the Regional Emission inventory in ASia (REAS) version 2 (REASv2, Kurokawa et al., 2013) was used. REASv2 provided a 0.25° × 0.25° resolution of anthropogenic emissions for the base years of 2000–2008 with monthly variations. They provided nine sectors for PM_x_: (1) aviation, (2) domestic, (3) industry, (4) international navigation, (5) other transport, (6) power plants (nonpoint sources), (7) power plants (nonpoint sources in Japan), (8) power plants (large point sources), and (9) road transport. For Japan, three bottom‐up inventories were used. The Ministry of the Environment, Japan (MOEJ) PM_2.5_ emission inventory (which also included TSPs) PM2.5EI was mainly used (Japan Petroleum Energy Center (JPEC), 2016; Morikawa, 2017). PM2.5EI provided a 1 km × 1 km resolution of anthropogenic emissions for the base year of 2012 with monthly, hourly, and weekday/holiday variations. They originally provided 25 sectors for PM_x_ and combined them into 10 sectors as follows: (1) electricity industry, (2) heat supply and gas production, (3) nonmanufacturing industry (agriculture, forestry, fishery, mining, and building), (4) manufacturing (furniture and fitments), (5) manufacturing (pulp, paper, and paper craft), (6) manufacturing (chemical engineering), (7) manufacturing (petroleum and coal products), (8) manufacturing (ceramic, stone, and clay products), (9) manufacturing (iron and steel), (10) manufacturing (nonferrous metals and products), (11) manufacturing (fabricated metal products), (12) manufacturing (others), (13) domestic (house and office), (14) operating machine, (15) aviation, (16) incineration, (17) small burn, (18) field burn, (19) smoking, (20) cooking, (21) traffic exhaust (passenger cars), (22) traffic exhaust (bus), (23) traffic exhaust (light duty trucks), (24) traffic exhaust (heavy duty trucks), and (25) traffic exhaust (special use trucks). Note that only the diesel oil of the five road transport sectors (21–25) was considered for the semi‐bottom‐up inventory because the contributions of emission fluxes of PM_x_ from other gases (gasoline and liquefied petroleum gas) and diesel oil from other sectors (mini passenger cars, mini trucks, and motor cycles) are small (approximately 3.2%) in PM2.5EI. For the other sectors in Japan, the EAGrid anthropogenic emission inventory of Japan (Fukui et al., 2014; Kannari et al., 2007) with a 1 km × 1 km resolution and monthly, hourly, and weekday/holiday variations for the base year 2010 was used for the three sectors: (1) tire, (2) brake, and (3) navigation. The railway could be an important emitter of metals; however, there is no nationwide emission record available in Japan. The only information available was a survey of the TMG (2011b) over the Kanto region prefectures (Tokyo and six surrounding prefectures: Ibaraki, Tochigi, Gunma, Saitama, Chiba, and Kanagawa), which provided line sources of PM_x_ emissions from rail wear of the track, tire, brake, and trolley line, for the base year of 2008 with monthly, hourly, and weekday/Saturday/holiday variations.

**Table 2:** List of Bottom‐Up Emission Inventories Used in the Study

Region	Inventory	Resolution	Base year	Sectors for PM_x_
Asia	REASv2	0.25°, monthly	2000–2008	9
Japan	PM2.5EI	1 km, hourly, weekday/holiday, and monthly	2012	25
Japan	EAGrid	1 km, hourly, weekday/holiday, and monthly	2010	3 (tire, brake, navigation)
Kanto	TMG survey	Line sources, hourly, weekday/Saturday/Sunday/holiday	2008	1 (railway)

MD particles contain certain amounts of transition metals. Transition metal emissions associated with the Asian MD particles (or yellow sand) (hereafter called Asian dust or Kosa: yellow sand in Japanese) were obtained using the metal profiles of the Certified Reference Material of the National Institute for Environmental Studies of Japan (NIES CRM No. 30; Gobi Kosa) (Nishikawa et al., 2013). The emission of Asian dust particles is calculated inline by the numerical model as a function of land use, friction velocity, soil moisture, and snow cover (Kajino, Deushi, et al., 2019).

Table 3 summarizes the emission inventories developed in the study. Ten transition metals, which consumed DTT as presented by Charrier and Anastasio (2012), were selected, namely, Cu, Mn, Co, V, Ni, Pb, Fe, Zn, Cd, and Cr. Although Charrier and Anastasio (2012) provided the DTT consumption rates of each metal ion, the water solubility was not considered, and thus, only the total mass of elements was considered in the inventories. The emissions of 10 transition metals associated with anthropogenic PM_2.5_ and PM_10_ were available for the Asia, Japan, and Kanto regions. Note that the 1 km × 1 km resolution of the Japan inventories (PM2.5EI and EAGrid) was averaged over the 2 km × 2 km resolution, and the line source emission of the Kanto railway inventory was also allocated to the same resolution (2 km × 2 km). The same transition metals were included in the Asian dust emission, except for V and Cd, which were below the detection limits listed in NIES CRM No. 30. It should be noted that there was no size separation for the metals in Asian dust because the size distribution of Asian dust is represented by a single mode in the numerical model (Kajino, Deushi, et al., 2019). The maximum, average, and minimum estimates are provided for the Asia and Japan inventories, while unique values are provided for the Kanto Railway and Asian dust emissions.

**Table 3:** Emission Inventories of Transition Metals Developed in the Study

Region	Resolution	Species	Values	Sectors	Semi‐bottom‐up inventory
Asia	0.25°, monthly	Cu, Mn, Co, V, Ni, Pb, Fe, Zn, Cd, and Cr in PM_2.5_ and PM_10_	Max, Ave, and Min	9	TMI‐Asia v1.0^a^ (average value)
Japan	2 km, hourly, weekday/holiday, monthly	Cu, Mn, Co, V, Ni, Pb, Fe, Zn, Cd, and Cr in PM_2.5_ and PM_10_	Max, Ave, and Min	28	TMI‐Japan v1.0^a^ (average value)
Kanto	2 km, hourly, weekday/holiday, monthly	Cu, Mn, Co, V, Ni, Pb, Fe, Zn, Cd, and Cr in PM_2.5_ and PM_10_	Unique	1 (railway)	
^b^	^b^	Cu, Mn, Co, Ni, Pb, Fe, Zn, and Cr	Unique	1 (Asian dust)	

^a^ Co and Cd were not included because they were not evaluated by the observation data in the study.

^b^ Up to simulation settings.

As presented in section 4.1, the simulations that used the average estimates of the inventories matched best among the three estimates. Therefore, in this study, we published new emission inventories: the semi‐bottom‐up inventory developed based on REASv2 with the average emission factors of SPECIATE v4.4 is referred to as the Transition Metal Inventory (TMI)‐Asia v1.0 (resolution: 0.25° × 0.25°, profile: monthly, area: Asia, sectors: 9), and the semi‐bottom‐up inventory developed based on the PM2.5EI, EAGrid, and TMG survey with the average emission factors of SPECIATE v4.4 is referred to as TMI‐Japan v1.0 (resolution: 2 km, profile: hourly, weekday/holiday, and monthly, area: Japan [Kanto for railway], sectors 29).

The volcanic metal emissions were not considered. Edmonds et al. (2018) suggested that arc volcanoes emit substantial amounts of metals associated with aerosols compared to the emissions of hotspot volcanoes. Among the elements listed above, they reported large emissions of Cu, Zn, Pb, and Cd in the ranges of 10^1^–10^4^ kg/d/volcano. There are several very active arc volcanoes located in Japan, and thus, the contributions of volcanic emissions to the surface air concentrations should be quantified in the future.

### 2.2 Numerical Model and Simulation Setup

The Japan Meteorological Agency's (JMA) regional‐scale meteorology‐chemistry model (NHM‐Chem; Kajino, Deushi, et al. (2019)) was used in the study. The offline meteorology‐chemistry coupling version was used. It considers full tropospheric photochemical reactions, aerosol microphysics, and dry and wet removal processes, including in‐cloud and below‐cloud scavenging. It also provides a simple transport model version without considering chemical reactions and a part of aerosol microphysics, such as new particle formation, condensation, and coagulation, which are strongly related to chemical reactions. This version was developed for the simulations of dispersion and deposition of species, which can be assumed to be chemically inert for the atmospheric life time scale. Currently, two options are available for radionuclides (Kajino, Sekiyama, et al., 2019) and transition metals (current study). The transition metal version employs three aerosol categories (or modes): SUB (anthropogenic submicron particles), COR (anthropogenic coarse mode particles), and MD. The triple‐moment modal approach with log‐normal size distribution was employed to simulate the size distribution of the metal‐bearing aerosols. At the point of emission, the number equivalent geometric mean aerodynamic dry diameters, standard deviations, and particle densities of SUB, COR, and MD are assumed to be 0.1, 2.0, and 2.0 μm; 1.7, 2.0, and 2.0; and 1.7 × 10^3^, 2.0 × 10^3^, and 2.0 × 10^3^ kg m^−3^, respectively. These parameters change only due to transport and removal processes. The prescribed hygroscopicity *κ* is assumed to be 0.3, 0.1, and 0.0 for SUB, COR, and MD, respectively, for the calculations of hygroscopic growth and cloud condensation nucleation (CCN) activity in removal processes.

**Figure 1:**
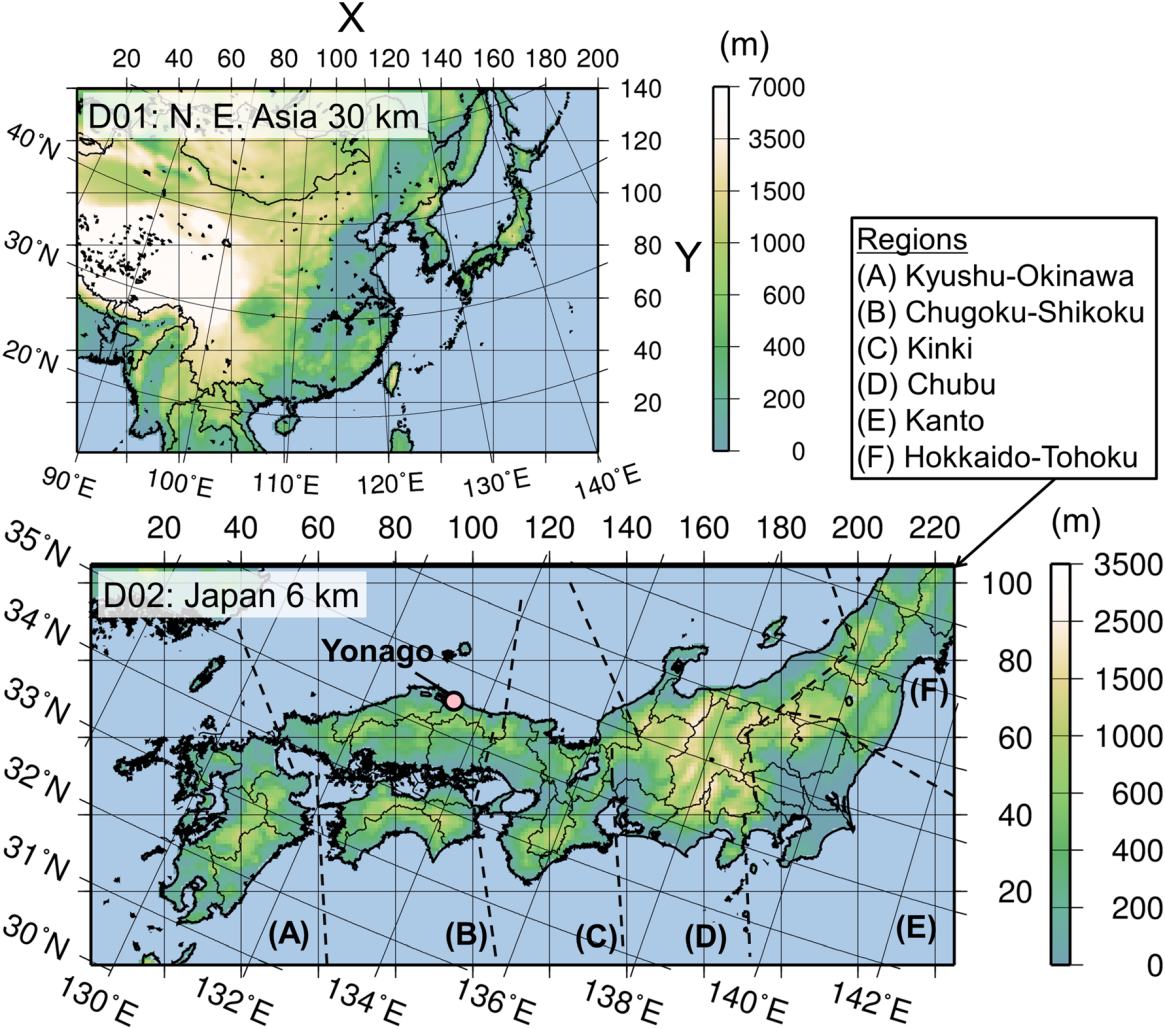
Model domains covering East Asia (Domain 1, Δ*x* = 30 km) and Japan (Domain 2, Δ*x* = 6 km) with terrestrial elevations. The observation site (Yonago) and regional names in Japan are defined in the study. The national borders and prefecture borders are depicted in D01 and D02, respectively.

Figure 1 shows the model domains. Domain 1 (D01) covering northeast Asian countries with a grid spacing of 30 km with 200 × 140 grid cells on the Lambert conformal conic projection, and Domain 2 (D02) covers the Kyushu, Shikoku, and Honshu (only Chugoku, Kinki, Chubu, Kanto, and a part of Tohoku regions) islands of Japan with a grid spacing of 6 km with 226 × 106 grid cells on the Lambert conformal conic projection. D02 covers densely populated areas of Japan, which are affected by massive long‐range transport from the Asian continent, and thus, finer grid resolution is necessary to accurately evaluate the domestic and transboundary contributions to the surface concentrations. The semi‐bottom‐up inventories based on REASv2 and those based on the PM2.5EI, EAGrid, and TMG‐ surveys were used for the simulations of D01 and D02, respectively. For the meteorological simulations, JRA‐55 global reanalysis (Kobayashi et al., 2015) (1.25° × 1.25°, 6 h) was used for the initial and boundary conditions. Spectral nudging was applied to constrain the simulated meteorological fields for the analysis. For the offline‐coupling version, the vertical coordinates were different for the meteorology and chemistry parts. Vertically, there are 38 levels up to 22,055 m above sea level (a.s.l.) and 40 levels up to 18,000 m a.s.l. for the meteorology and chemistry parts, respectively. Because there are no global inventories and models available for the targeted species, a zero concentration value was assumed for the boundary conditions of D01. JMA's Meso‐Regional Objective Analysis (MANAL, 5 km × 5 km, 3 h) was used for the initial and boundary conditions of the meteorology part and for the spectral nudging of D02. There are 48 vertical levels up to 21,801 m a.s.l. and 19 levels up to 10,000 m a.s.l. for the meteorology and chemistry parts, respectively. The D01 result was used for the boundary concentration of D02 in the chemistry part, with a temporal resolution of 1 h. The simulation period was the entire year of 2013, with a spin‐up period of 5 days.

To estimate the domestic contribution to the simulated concentrations, the brute‐force method (e.g., Bartinicki, 1999) was used in this study.

(3) $$\frac{C_{k}}{C}=\frac{\left(C-C\left[E_{k}\times 80\%\right]\right)\times 5}{C},$$

where *C*, *C*_*k*_, and *C*[*E*_*k*_ × 80%] indicate the simulated surface air concentration, concentration originating in source region *k*, and concentration simulated by 80% emission of region *k*, respectively. The domestic contribution was derived as 1 − *C*_*k*_/*C*, with *k* outside the country.

## 3 Observation Data

### 3.1 Nationwide Seasonal Monitoring of PM_2.5_ Elements in Japan

The nationwide seasonal monitoring of PM_2.5_ inorganic elements conducted by MOEJ was used to evaluate the simulated seasonal mean PM_2.5_ values (SUB) as well as their spatial distributions. Daily PM_2.5_ sampling and chemical composition analyses were conducted for 2 weeks in the four seasons at approximately 150 stations in Japan. The data sets are available at http://www.env.go.jp/air/osen/pm/monitoring.html (last accessed: 15 April 2019) for each Japanese fiscal year, which start on 1 April. To compare the simulation results for the year 2013, the winter samples for the fiscal year 2012 and the spring, summer, and autumn samples for the fiscal year 2013 were used for the model evaluation. The monitoring sites were categorized into “roadside,” “general,” and “background” sites, and there were 101, 32, and 19 sites, respectively, for the fiscal year 2013. The technical manual of the measurements is available at https://www.env.go.jp/air/osen/pm/ca/manual/manual-3.pdf (last accessed: 15 April 2019) (in Japanese). The measurement protocols depend on the sites, but they are very similar. Aerosols were collected by PM_2.5_ samplers on polytetrafluoroethylene (PTFE) filters with flow rates of 15–30 L min^−1^, and the inorganic elements were measured using inductively coupled plasma mass spectrometry (ICP‐MS) at approximately 90% of the sites or using X‐ray fluorescence (XRF) methods at the other 10% of the sites. The list of sites and their measurement protocols are available in the measurement data.

### 3.2 Long‐Term Continuous Measurement of TSP Elements in Yonago, Japan

The continuous daily measurement data of TSP at Yonago City, Tottori Prefecture, Japan (Figure 1), were used for the evaluation of the simulated PM_10_ values (SUB + COR + MD) and their temporal variations. The measurement period was from March to December 2013. Aerosols were collected by a TSP sampler (MCAS‐03, Murata Keisokuki Service Co. Ltd.) on PTFE filters (Whatman, PM_2.5_ Air Monitoring PTFE Membrane Filter, 46.2 mm φ) with a flow rate of 30 L min^−1^. The sampler was situated on a rooftop terrace of the building of Faculty of Medicine, Tottori University (35.43°N, 133.33°E), approximately 20 m above ground level (a.g.l.). The inorganic elements were analyzed using the energy‐dispersed XRF (EDXL300, Rigaku Corp., Japan) coupled with the fundamental parameter (FP) quantification method (EDXRF‐FP), which has been developed and evaluated by Okuda et al. (2013, 2014).

## 4 Results and Discussion

In this section, among the 10 metals, only the top five metals in the DTT consumption rate per unit of element mass in the air, namely, Cu, Mn, Fe, V, and Ni (Charrier & Anastasio, 2012; Fujitani et al., 2017), are presented and discussed. Cu is the most important element among the transition metals based on reagent experiments of both Charrier and Anastasio (2012) and Fujitani et al. (2017), followed by Mn and then Fe for Charrier and Anastasio (2012) and Fe and then Ni for Fujitani et al. (2017), respectively. Pb, Zn, and Cr are presented and discussed in Appendix A. Co and Cd are not presented in the study because the observed Co concentrations were very low, almost below the detection limits in Japan, and because no observation data were available for Cd.

As presented later, the average estimates of the semi‐bottom‐up inventories based on the REASv2 and PM2.5EI/EAGrid/TMG survey are published and referred to as TMI‐Asia v1.0 and TMI‐Japan v1.0, respectively. However, Co and Cd are not included in the published inventories.

### 4.1 Comparison With Observations

For the comparisons presented in this section, both D01 and D02 simulations are compared against observations for the assessment of the semi‐bottom‐up inventories based on REASv2 and PM2.5EI/EAGrid/TMG survey, respectively.

**Figure 2:**
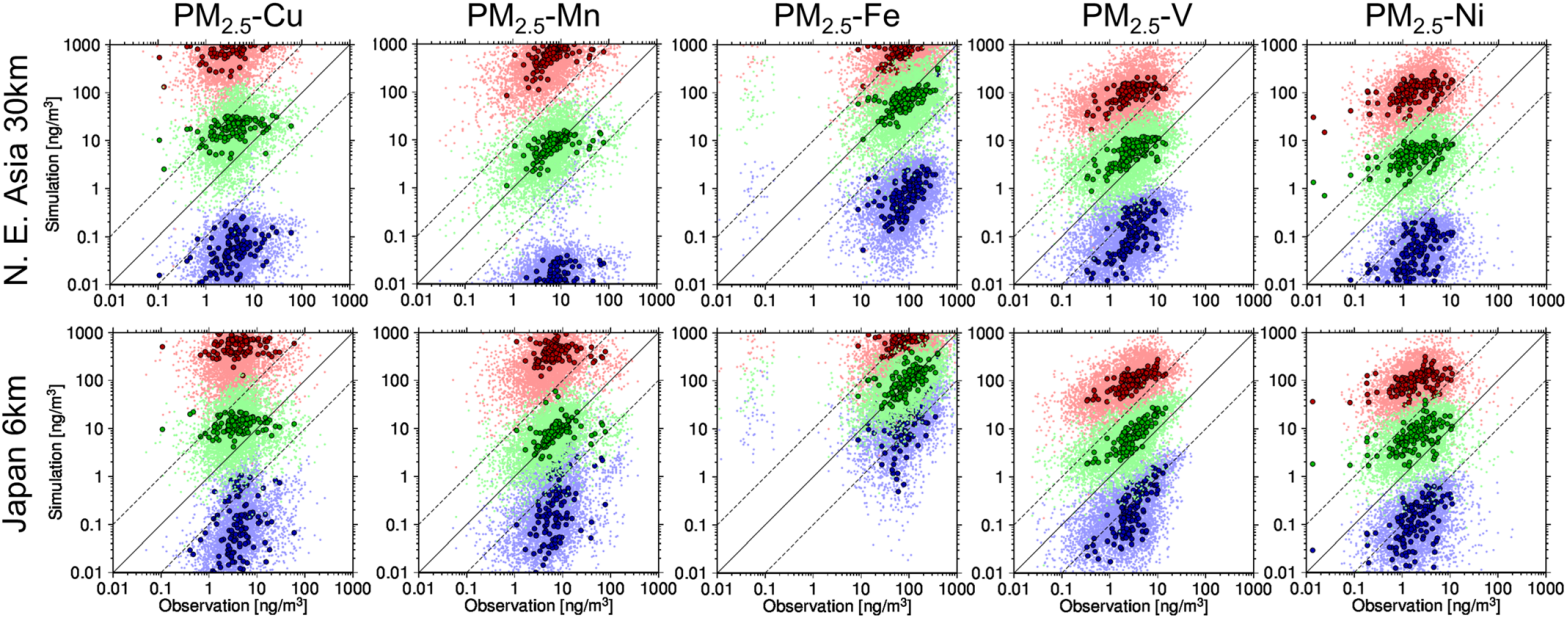
Scatter diagrams between the simulated (top: D01 with the REASv2 base inventory; bottom: D02 with PM2.5EI/EAGrid/TMG survey base inventory) and the observed daily (small dots with light colors) and annual mean (larger dots with thick colors; 8 weeks of mean of daily values, 2 weeks in each season) (left to right) Cu, Mn, Fe, V, and Ni of PM_2.5_ at the MOEJ monitoring stations in Japan. The red, green, and blue dots indicate the simulations with the maximum, average, and minimum estimates, respectively. The solid line indicates the 1:1 line, and the two dashed lines indicate the boundaries of the factor of 10.

Figure 2 compares the simulated and observed Cu, Mn, Fe, V, and Ni in the PM_2.5_ at the MOEJ monitoring stations in Japan. Although some MD particles could contribute to the PM_2.5_ measurements, the simulated anthropogenic fine mode elements (SUB) were compared with the observations. The simulations with the semi‐bottom‐up inventories were successful because the observation data were settled within the ranges of the maximum and minimum estimates of the simulations. Among the three estimates, the maximum and minimum simulation results significantly overestimated and underestimated the observations, respectively, and the average result matched best with the observation.

The statistical metrics used for the comparison are summarized in Table 4, which shows only the comparison for the annual mean values (i.e., mean of 8 weeks based on daily values from 8 weeks in each season); thus, the table shows the spatial correlations between the simulation and observation. The best spatial correlation was obtained for V (*R* = 0.86 and 0.52), followed by that for Fe (*R* = 0.52 and 0.64) and Ni (*R* = 0.42 and 0.48). There were no spatial correlations for Cu (*R* = 0.0 and 0.23) and Mn (*R* = 0.086 and 0.31). Even though the spatial correlations were lower for Cu and Mn, higher temporal correlations were obtained for specific sites if the 8‐week‐in‐a‐year measurement successfully captured the peak concentration events due to meteorological conditions, which could be well reproduced by the simulation. For example, the best temporal correlation coefficient was obtained at the Shibata site in Niigata prefecture (station ID: 115206001), with a value of *R* = 0.86. Nevertheless, it should be noted here that the emission factors selected for Cu and Mn should be improved. In terms of the simulation to observation median ratio (*Sim:Obs*), the estimation and simulation of Mn and Fe were successful, and more than 80% and 90% of the simulated values were within factors of two and five, respectively, while those of Cu, V, and Ni were overestimated. Nevertheless, approximately 70–90% of the simulated values were within factors of five for Cu (D02 only), V, and Ni.

**Table 4:** Statistical Metrics for Comparison of the Simulated (With the Average Estimates of the Inventory) and Observed Annual Mean (8 weeks of mean of daily values, 2 weeks in each season) concentrations in the PM_2.5_ at the MOEJ monitoring stations in Japan

	*N* ^a^	Obs. Med.^b^	*Sim:Obs* ^c^	*R* ^d^	*Fa2* ^e^	*Fa5* ^f^
Unit		ng m^−3^	‐	‐	‐	‐
Cu	120 (127)	3.86 (3.75)	3.3 (5.4)	0.0 (0.23)	0.20 (0.11)	0.78 (0.48)
Mn	121 (130)	6.96 (6.72)	1.2 (1.3)	0.086 (0.31)	0.83 (0.82)	0.92 (0.96)
Fe	133 (142)	94.4 (90.6)	1.1 (0.85)	0.52 (0.64)	0.86 (0.82)	0.96 (0.98)
V	138 (147)	2.77 (2.74)	2.6 (2.1)	0.86 (0.52)	0.29 (0.46)	0.97 (0.94)
Ni	135 (144)	1.75 (1.71)	3.7 (3.1)	0.42 (0.48)	0.16 (0.26)	0.67 (0.79)

*Note*. Values without and with brackets are of the D02 and D01 simulations, respectively.

^a^ Number of available data (equivalent to number of stations in the model domains).

^b^ Median of observation data.

^c^ Simulation to observation median ratio.

^d^ Correlation coefficient.

^e^ Fraction of simulated values within a factor of two of the observed values.

^f^ Fraction of simulated values within a factor of five of the observed values.

**Figure 3:**
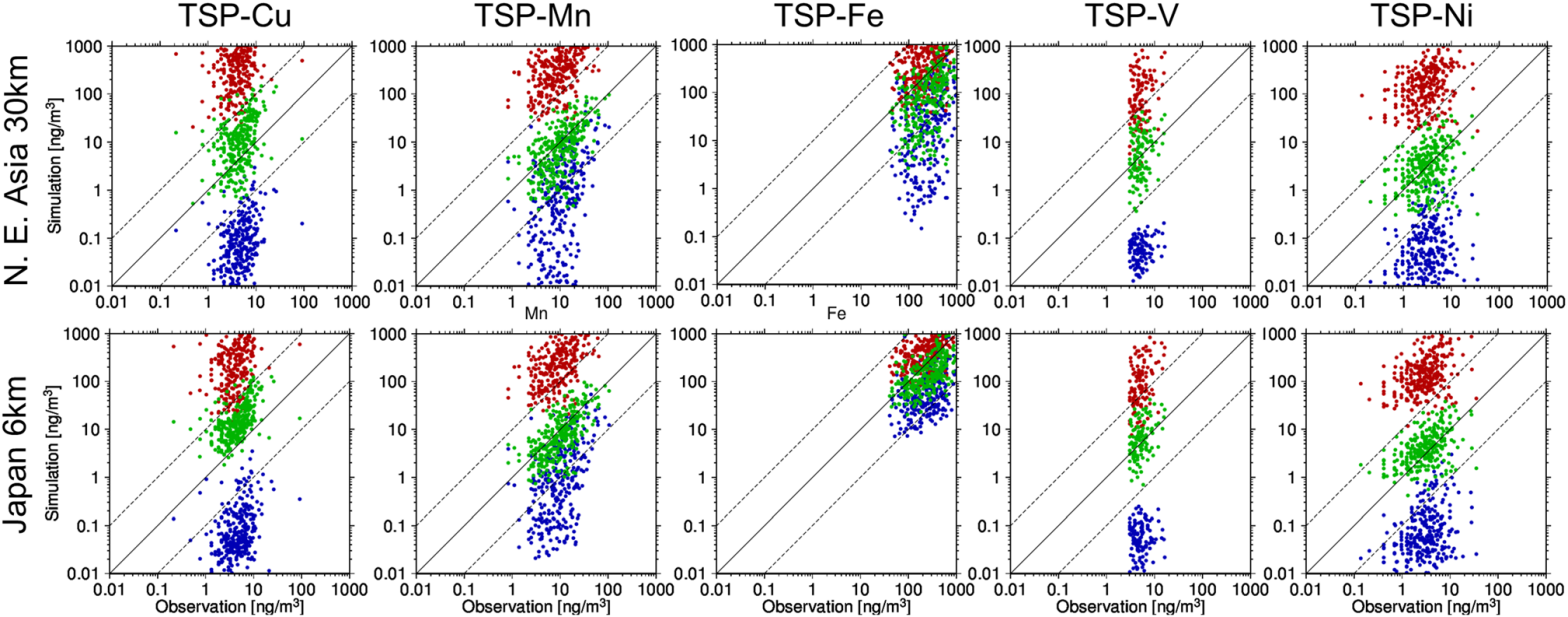
Same as Figure 2 but for the daily TSP measurement at Yonago.

Figure 3 compares the simulated and observed Cu, Mn, Fe, V, and Ni in the TSP at the Yonago site. The simulated anthropogenic fine and coarse mode elements (SUB + COR) and those from MD were compared against the observations. The same conclusion as that made from the PM_2.5_ comparison was obtained: the observed values were settled within the three estimates, and the average was the best among the three. The statistical metrics are summarized in Table 5. Table 4 shows the spatial agreement between the simulation and observation, while Table 5 indicates the temporal agreement. However, because long‐term continuous measurements are available at the site and there are no large emission source regions near the site, the air masses from different source regions could be evaluated by the comparison. In other words, the comparison can validate not only local metal emissions but also emissions from different sources transported to the site. As presented later in section 4.4, the long‐term variation in the surface concentration at Yonago could be dominantly affected by transboundary air pollution (e.g., Onishi et al., 2018). The site is also under the influence of massive transport of Asian dust in spring (section 4.4; e.g., Kajino, Deushi, et al. (2019)). These differences contributed to the differences in values and trends between Table 5 and Table 4. There were higher temporal correlations for Fe and Mn (*R* = 0.6–0.7) than for other metals (*R* = 0.3–0.4). The contributions of Asian dust were large for Fe and Mn; thus, the successful simulation of the Asian dust transport in spring at Yonago could be a reason for the higher *R*, while the *Sim:Obs* values were lower (0.36–0.79). With respect to *Sim:Obs*, better performances were obtained for V and Ni, with the lowest influences from the Asian dust particles. Similar to the comparison for PM_2.5_, the simulated Cu in the TSP overestimated the observation.

**Table 5:** Same as Table 4 but for the Daily Metal Concentrations in TSP at Yonago

	*N* ^a^	Obs. Med.	*Sim:Obs*	*R*	*Fa2*	*Fa5*
Unit	‐	ng m^−3^	‐	‐	‐	‐
Cu	297	4.65	2.8 (2.2)	0.31 (0.30)	0.28 (0.35)	0.76 (0.78)
Mn	296	9.96	0.70 (0.79)	0.67 (0.68)	0.61 (0.56)	0.94 (0.91)
Fe	296	277	0.51 (0.36)	0.61 (0.64)	0.53 (0.32)	0.93 (0.68)
V	117	4.98	1.2 (0.99)	0.42 (0.31)	0.63 (0.50)	0.97 (0.86)
Ni	283	3.03	1.3 (0.99)	0.40 (0.33)	0.59 (0.52)	0.92 (0.86)

^a^ Number of available data (equivalent to number of days when the data are available).

Even though there were still discrepancies between the observed and the simulated transition metals, the semi‐bottom‐up inventories with the average estimates were published in this study and are referred to as TMI‐Asia v1.0 and TMI‐Japan v1.0. No further improvement in the emission flux has been made thus far due to the following reasons: the discrepancies shown in Figures 2 and 3 and Tables 4 and 5 originated from uncertainties in both the emission inventory and the numerical simulation, but they were not inseparable. There were enough comprehensive measurement data sets available in Japan to validate the domestic emissions, but there were not as many data available in other Asian countries, and thus, the emissions from the other countries and the transboundary fractions to the surface concentration in Japan could not be validated. There are two main ways to improve the emission fluxes for future development: a bottom‐up approach to directly improve the emission factors or metal profiles after the detailed investigations for each sector and country and a top‐down approach to constrain the emission factors, metal profiles, or emission fluxes using field observations as done by Ying et al. (2018). Ying et al. (2018) improved the semi‐bottom‐up emission inventory by modification of metal profiles using a transport model and field measurements in Beijing. The modified inventory was validated against the independent measurements in Nanjing and Chengdu. However, the uncertainty in transport modeling has not been taken into account in the estimation. An inverse modeling (e.g., Ying et al., 2018; Yumimoto et al., 2016) using multimodel ensembles (e.g., Itahashi et al., 2020; Kajino, Sekiyama, et al., 2019; Li et al., 2019) may be a useful way to overcome the issue, because the multimodel ensemble simulations can reduce uncertainty in transport modeling.

### 4.2 Transition Metals Inventories v1.0

**Figure 4:**
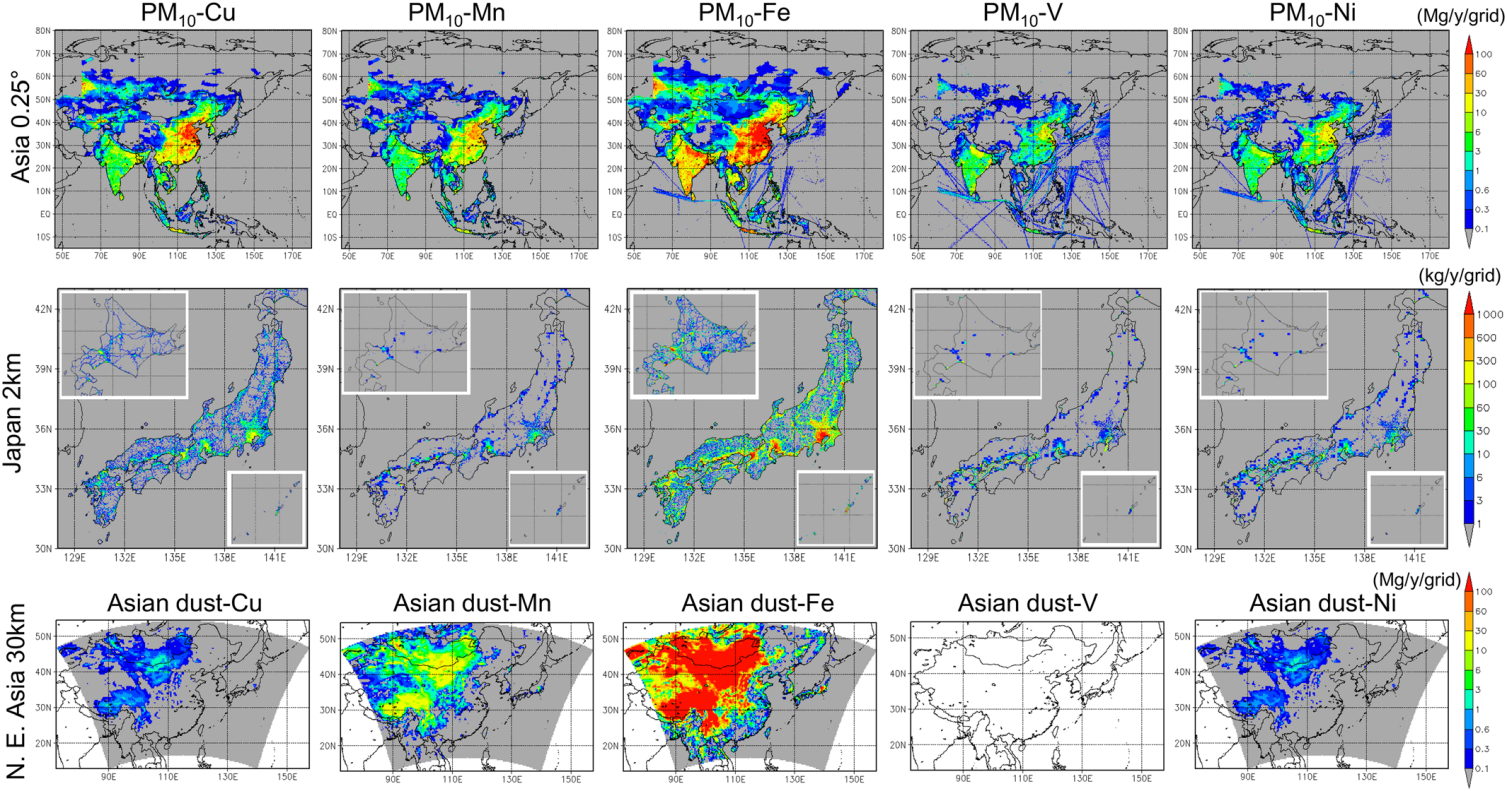
The spatial distribution of annual total emission fluxes of (left to right) Cu, Mn, Fe, V, and Ni in PM_10_ of (top to bottom) anthropogenic fluxes in Asia (TMI‐Asia v1.0; 0.25° × 0.25° for the base year 2008; Mg y^−1^ grid^−1^), anthropogenic fluxes in Japan (TMI‐Japan v1.0; 2 km × 2 km for the base year 2010; kg y^−1^ grid^−1^), and fluxes associated with Asian dust particles over D01 (30 km × 30 km on the Lambert conformal conic projection for the year 2013; Mg y^−1^ grid^−1^).

The spatial distributions of the annual total emission fluxes of the five transition metals of TMI‐Asia, TMI‐Japan, and Asian dust particles are illustrated in Figure 4. The areal total amounts together with *Sim:Obs* are listed in Table 6.

**Table 6:** Emission Amounts of Areal Total Transition Metals Developed in the Study and the Simulation to the Observation Median Ratio of PM_2.5_ at the MOEJ Stations

	TMI‐Asia^a^	TMI‐Japan^b^	Asian dust^c^	*Sim:Obs* ^d^	*Sim:Obs* ^d^
Regions	Total/D01/Japan^e^	Japan	D01	D01	D02
Unit	Gg y^−1^	Gg y^−1^	Gg y^−1^	‐	‐
Cu	233/187/0.562	0.643	2.18	5.4	3.3
Mn	130/106/0.303	0.891	43.4	1.3	1.2
Fe	904/668/2.26	7.95	2,460	0.85	1.1
V	72.3/49.8/0.212	0.414	0.0	2.1	2.6
Ni	75.9/52.4/0.185	0.382	1.86	3.1	3.7

^a^ Anthropogenic PM_10_ for the base year of 2008.

^b^ Anthropogenic PM_10_ for the base year of 2010.

^c^ For the simulation year, 2013.

^d^ Table 4.

^e^ Only over land: accumulated only over the grids if the land mask of Japan exceeds 15% of the REASv2 0.25° × 0.25° grid.

The annual total emission of Fe over all of Asia was the largest, at 904 Gg y^−1^, among which approximately 70% was emitted from D01 (668 Gg y^−1^). Iron (Fe) from Asian dust was 3.5 times greater than that from anthropogenic origin over D01 (2,460 Gg y^−1^). The anthropogenic Fe from Japan was 2.5% of that from Asia (2.26 Gg y^−1^), but the value varied by 3.5‐fold (2.26 and 7.95 Gg y^−1^) depending on the selection of the bottom‐up inventory (e.g., its emission amounts and available sectors). The second largest amount was for Cu (233 Gg y^−1^) and then Mn (130 Gg y^−1^); however, Mn may be the second largest because the Cu emission may be overestimated. The amounts of V and Ni are similar with each other. The areal fractions (total/D01/Japan) of other metals in TMI‐Asia are similar to those of Fe. The total amounts of TMI‐Japan are generally larger than those of TMI‐Asia but consistent with each other. Except for Fe, the anthropogenic emission of Mn was slightly larger than that from Asian dust (2.4 times), while the anthropogenic Cu, V, and Ni emissions were much larger than those from Asian dust.

### 4.3 Source Contributions of Transition Metals Emissions in TMI‐Japan v1.0

**Figure 5:**
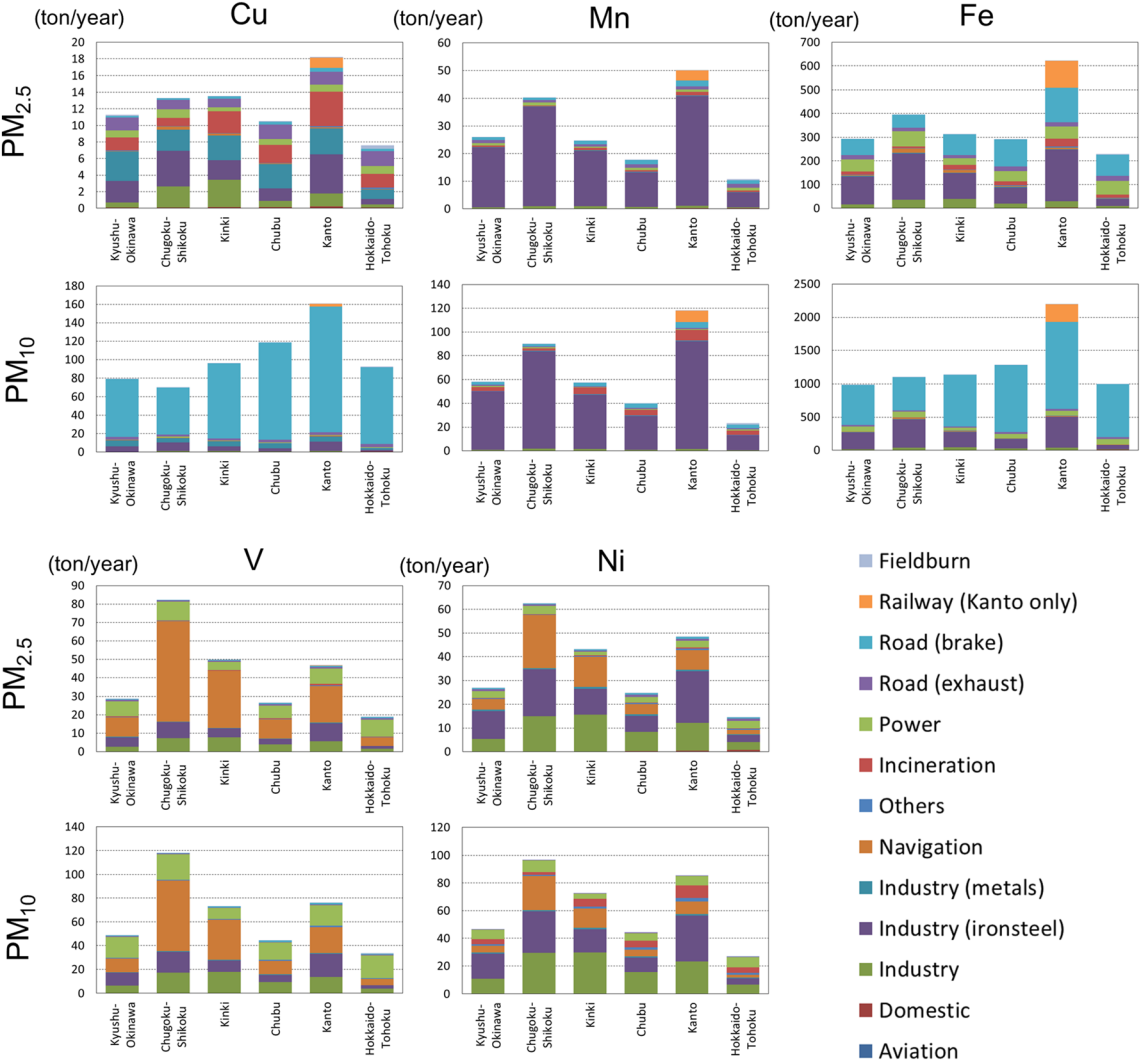
Total emission amounts with the contributions of each emission sector for transition metals of TMI‐Japan (v1.0) (and thus anthropogenic only) for the six regions, as presented in Figure 1. Note that TMI‐Japan (v1.0) provides railway emissions only from Kanto.

The contributions of each emission sector to the five transition metals in PM_2.5_ and PM_10_ provided by TMI‐Japan (and thus anthropogenic only) are presented in Figure 5.

Emissions of metal‐bearing particles are associated with coal combustions (“power plants” and “iron‐steel industry”), metal fume (“iron‐steel industry” and “metal industry other than iron‐steel”), frictional wear (“road brake” and “railway”), tailpipe exhaust of fuel additives (“road exhaust”), combustion of residual fuel oil (“navigation”), and refuse incineration of metal containing materials (“incineration”) (Abbasi et al., 2013; Dall'Osto et al., 2008; Hagino et al., 2016; Nagajyoti et al., 2010; Schauer et al., 2006).

The most important sector for PM_10_‐Cu was “road brake,” which accounted for more than 80%, whereas for PM_2.5_‐Cu, other sectors such as “other industry (nonmetals),” “iron‐steel industry,” “metal industry other than iron‐steel,” “incineration,” “power plants,” “road exhaust,” and “railway” (Kanto only) had almost equal contributions (from only a few percent up to 10–20%). However, it should be noted that the size distribution of the current inventory has not yet been evaluated. In fact, a recent laboratory experiment (Hagino et al., 2016) showed that most brake wear particles existed in the fine mode, i.e., PM_2.5_. The size apportionment of the emission inventory certainly needs further improvement.

The “iron‐steel industry” accounted for 60–90% for both PM_10_‐Mn and PM_2.5_‐Mn, followed by “incineration,” “road brake,” and “railway” (Kanto only) for up to 10%.

For PM_10_‐Fe, “road brake” was the most important sector, which is similar to PM_10_‐Cu, but “iron‐steel industry” (10–40%) and “railway” (10% in Kanto) were also contributed. For PM_2.5_‐Fe, “iron‐steel industry” was the most important sector (20–50%), followed by “road brake” (20–40%) and “railway” (20% in Kanto), and then “power plants” (10–20%).

**Figure A4:**
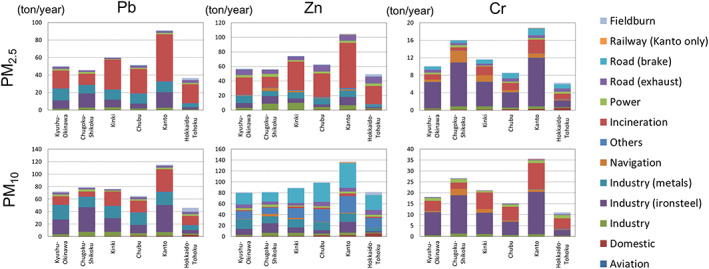
Total emission amounts with the contributions of each emission sector for Pb, Zn, and Cr of TMI‐Japan (v1.0) (thus, anthropogenic only) for all of Japan and the six regions, as presented in Figure 1. Note that TMI‐Japan (v1.0) provides railway emissions from only Kanto.

Nickel could also be an important DTT consumer based on an experiment of Fujitani et al. (2017). The source contributions of V and Ni were similar. The contributions of PM_2.5_ and PM_10_ were similar with each other, too. The total emissions from “navigation” were largest in Chugoku‐Shikoku for V and Ni, whereas the total amounts in the most populated area Kanto were largest in the other elements, such as Cu, Mn, and Fe (Pb, Zn, and Cr, too, as presented later in Figure A4). This result was due to the heavy fuel oil consumption from vessels in the Seto Inland Sea and industrial factories by the coast. The Seto Inland Sea, surrounded by the Chugoku and Shikoku regions, is a major rout of vessels in Japan, and large industrial regions are located along the coast. The major contributors for V and Ni were “navigation” (10–70%), “other industry (nonmetals)” (10–40%), and “power plants” (10–50%).

### 4.4 Horizontal Distribution of Surface Concentrations of Transition Metals

**Figure 6:**
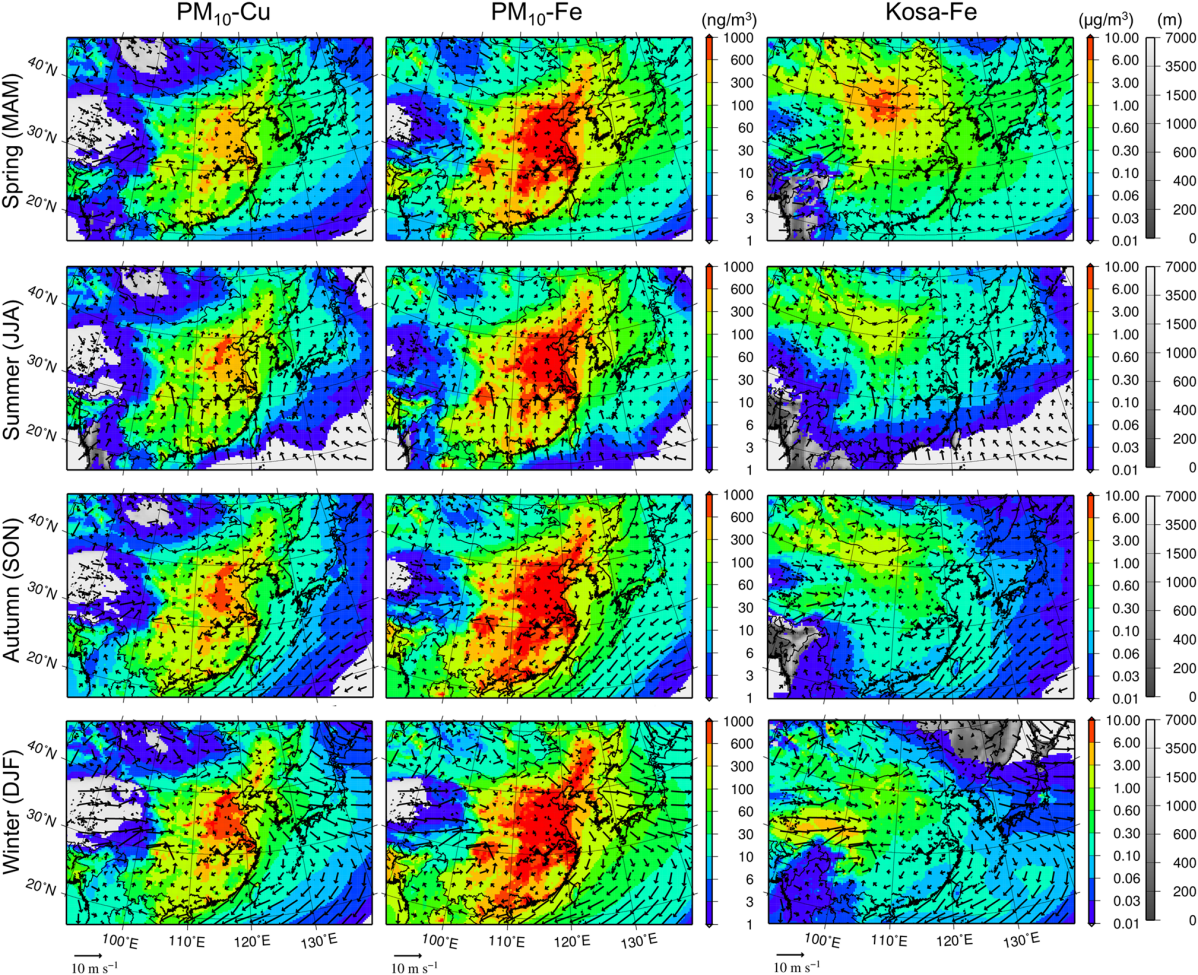
Seasonal mean surface air concentrations of (left to right) anthropogenic PM_10_‐Cu, anthropogenic PM_10_‐Fe, and Fe in Asian dust in (top to bottom) spring, summer, autumn, and winter of 2013 with surface wind vectors over D01. Note that the color bar of Asian dust Fe is one order larger than that for anthropogenic metals. The model terrestrial elevations are depicted under the shades.

**Figure 7:**
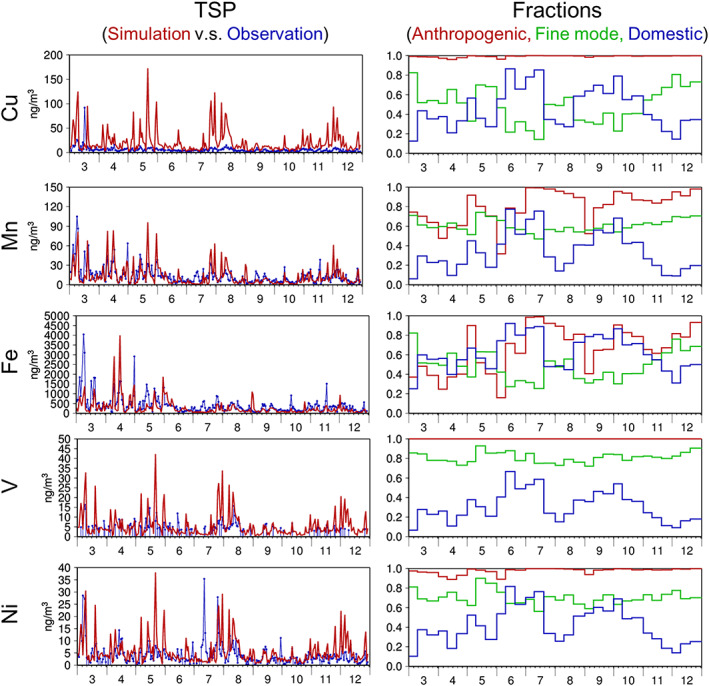
Temporal variations in (left) (red) simulated (D02) and (blue) observed (top to bottom) daily concentrations of Cu, Mn, Fe, V, and Ni in TSP and (right) 10‐day mean simulated (D02) fractions of (red) anthropogenic to total (anthropogenic + Asian dust), (green) anthropogenic fine mode to anthropogenic total (fine + coarse), and (blue) anthropogenic domestic PM_10_ to anthropogenic total (domestic and transboundary) PM_10_ at Yonago.

Figure 6 presents the seasonal mean surface mass concentrations of anthropogenic PM_10_‐Cu, anthropogenic PM_10_‐Fe and Fe in Asian dust in spring (MAM: March, April, and May), summer (JJA: June, July, and August), autumn (SON: September, October, and November), and winter (DJF: December, January, and February) for 2013. Figure 7 shows the time series of the simulated (PM_10_, D02) and observed (TSP) metal concentrations at Yonago, with the simulated (D02) fractions of anthropogenic vs. Asian dust, anthropogenic fine vs. anthropogenic coarse, and anthropogenic domestic vs. anthropogenic transboundary components. As mentioned in section 4.1, Yonago is located on the coast of the Sea of Japan, and there are no large emission source regions near the site. It is a suitable location to evaluate the long‐range transport of air pollutants from the Asian continent to Japan. Therefore, the seasonal variations presented in Figure 7 are consistently explained by Figure 6.

As shown in Figure 6, the surface concentrations of anthropogenic metals over central China are highest in winter due to the weakest convection. The long‐range transports from the Asian continent are predominant in spring and are associated with migrating disturbances. The surface concentrations in Japan are enhanced due to cyclonic post‐cold frontal transports and edge transports of anticyclones. The surface concentrations in Asia are strongly affected by seasonal monsoons in summer and in winter, the southerly wind of the Pacific High, and the northerly to westerly wind from the Siberian High, respectively. In summer, the long‐range transports from the continent were least significant, and domestic contributions generally became highest in Japan. Even in summer, as shown in Figure 7, surface concentrations became high from late July to early August, which was associated with the enhancement of long‐range transport. The stationary front prevails over central Japan, and the westerlies along with the front transported the contaminated air mass. Heavy precipitation occurred and was associated with the front; thus, certain amounts of aerosols could be scavenged in the presence of precipitation. In the autumn, the mean surface wind fields were primarily affected by the Yangtze High. In winter, the long‐range transports were predominantly associated with the northerly to westerly wind. The surface concentrations and the transboundary contributions were enhanced from late November (a part of autumn) to early December at Yonago due to the winter monsoon (Figure 7).

Massive Asian dust emissions occurred in arid and semiarid regions, such as in western and northern China, and the emission flux was generally proportional to the surface wind speed. The snow cover and soil wetness significantly suppressed the emission flux of Asian dust. Consequently, the emission and long‐range transport of Asian dust were highest in spring (Figure 6), and the surface concentrations of TSP‐Fe (TSP‐Mn as well) at Yonago were highest from March to May, as the anthropogenic contribution was lower (Figure 7). The Asian dust emission was large over the Tibetan Plateau in winter, but the metal compositions of the soil could be much different from those we assume based on Gobi Kosa (NIES CRM No. 30). The simulated metals in Asian dust from Tibet were not validated in this study.

There were no significant seasonal variations found in the fine mode fractions of the simulated anthropogenic PM_10_. Generally, the fine mode particles were transported longer than the coarse mode particles for the size ranges of approximately 1 μm because the dry deposition velocity and below‐cloud scavenging were larger (as larger inertia) and in‐cloud scavenging (as larger CCN activity due to the size effect) could be larger. Consequently, the simulated fine mode fractions and domestic contributions are inversely correlated at Yonago (Figure 7). The fine mode fractions of V and Ni are larger than those of Cu, Mn, and Fe. There were not many size‐resolved measurements made, but the results were somewhat consistent with the detailed measurements conducted in Yokohama, Japan (Okuda et al., 2007); specifically, the medians of the PM_2.5_/TSP ratios of V were almost uniform, and those of Mn, Ni, Cu, and Fe were approximately 0.8, 0.6, 0.5, and 0.4, respectively. Although the size distribution was not the main focus of the current study, the simulated size distribution of metals should be evaluated in future research.

## 5 Conclusions

Anthropogenic emission inventories of transition metals in Asia (TMI‐Asia, v1.0; 0.25°, monthly, 2000–2008, nine sectors) and Japan (TMI‐Japan, v1.0; 2 km, hourly, 2012, 29 sectors) were developed in this study, based on the bottom‐up inventories of REASv2 and PM2.5EI/EAGrid/TMG survey, respectively, and the average emission factors of SPECIATE v4.4 in each sector. The metal emissions from railways are available only in the Kanto Area of Japan, which is the most populated area and includes the Tokyo Metropolitan Area, for the base year of 2008. Toward the simulation of aerosol OP as a final goal, as a first step, 10 OP active transition metals as quantified by Charrier and Anastasio (2012) using the DTT (dithiothreitol) assay (Kumagai et al., 2002) were selected, namely, Cn, Mn, Co, V, Ni, Pb, Fe, Zn, Cd, and Cr in PM_2.5_ and PM_10_.

The emission inventories were evaluated by comparing the observations and simulations for the full year of 2013. NHM‐Chem was used for the simulation, with horizontal resolutions of 30 and 6 km over East Asia and Japan, for the evaluation of TMI‐Asia (v1.0) and TMI‐Japan (v1.0), respectively. Two observations were used: a nationwide seasonal PM_2.5_ monitoring conducted by MOEJ and a long‐term continuous TSP measurement at Yonago, Japan. Metals in Asian dust were also considered in the simulation and compared with the measured values. Cobalt (Co) and Cd were not evaluated due to the measured surface concentrations being too low. Our simulation was successful: the observations of all elements were well within the ranges of simulations with maximum and minimum emission estimations, and the simulations with the average emission estimations (=TMI v1.0) were closest to the observations. Most of the simulated elements generally agreed with the observations, with approximately 0.6 of the correlation coefficient (*R*) and approximately 0.5 and 0.9 of fractions of simulated values within factors of two and five of the observed values (*Fa2* and *Fa5*), respectively. Some of the simulated elements were significantly overestimated, such as Cu, V, Ni, Pb, and Cr, compared with the nationwide PM_2.5_ measurement (2–6 of the simulated to observed median ratios, *Sim:Obs*) and Cu and Pb compared with the TSP measurement at Yonago (2–3 of *Sim:Obs*).

The source contributions of anthropogenic transition metals emission in TMI‐Japan v1.0 were summarized. Road brakes and iron‐steel industry are primary sources, followed by other metal industry, navigation, incineration, power plants, and railway. The current estimation provides the effective strategies on emission reduction of DTT active elements: for example, abatement of Cu and other elements in brake pad could reduce its emission by 80%; filtration of particles emitted from iron‐steel industry and refinement of fuel for ships will efficiently reduce their metal emissions. Not presented in the study, but the effects of emission reductions on changes in the surface concentrations could be quantitatively assessed in the current emission inventory—transport modeling framework. However, the effectiveness of these emission reductions on oxidative stress that we receive cannot be assessed in the current framework.

Our final goal is accurate prediction of aerosol OP and its emission source contributions. The current study is a first step and substantial improvements, and developments must be made in the future. Future directions are itemized as follows:

1. The current emission inventory (v1.0) requires further updates. Improvement of metal profiles of the semi‐bottom‐up approach (Ying et al., 2018) or development of bottom‐up inventory (e.g., Tian et al., 2015) should be made. The current inventory was evaluated only by a single model and observations in Japan. A top‐down approach, such as inverse modeling (e.g., Yumimoto et al., 2016) using multiple models and international observations, would substantially improve the inventories.
2. The size apportionment of the current inventory needs further improvement. For example, “road brake” was the most important sector for PM_10_‐Cu, whereas a laboratory experiment (Hagino et al., 2016) showed that most brake wear particles existed in PM_2.5_. The size distributions and hygroscopicity of the host particles are important for the predictions of atmospheric behavior and lung deposition efficiency (e.g., Ching & Kajino, 2018); however, they were not fully assessed in this study.
3. Only total amounts of elements were considered in the study, but the chemical properties of elements, such as water solubility, are also important. The water solubility of elements should vary across emission sectors, and it also changes during long‐range transport, i.e., aerosol acidity makes metal elements water soluble (Ito et al., 2018).
4. Organics should be considered in the inventories as well as the numerical model. Quinones efficiently consume DTT. In addition to transition metals, modeling quinones is indispensable for the prediction of aerosol OP. Quinones could be very important in ROS production in cells because they overproduce hydrogen peroxide without being consumed themselves (Motoyama et al., 2009). Interactions between metals and organics in DTT (Yu et al., 2018) as well as in ROS generation (Fang et al., 2019) should be considered in the model.

## Conflict of Interest

The authors declare no conflicts of interest relevant to this study.

## Supporting information

Supporting Information S1

## Figures and Tables

**Figure A1:**
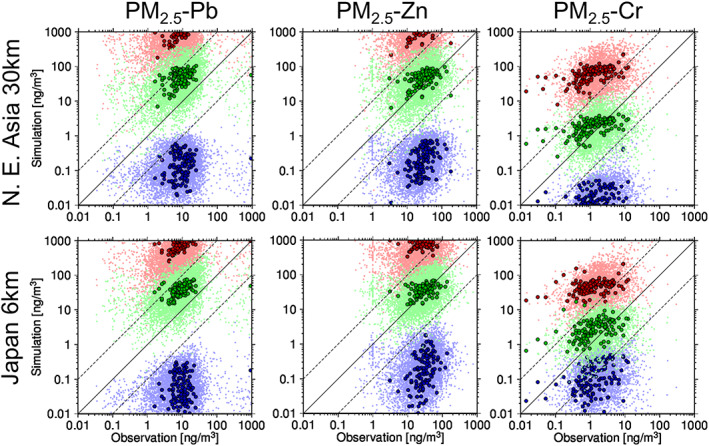
Same as Figure 2 but for Pb, Zn, and Cr.

**Figure A2:**
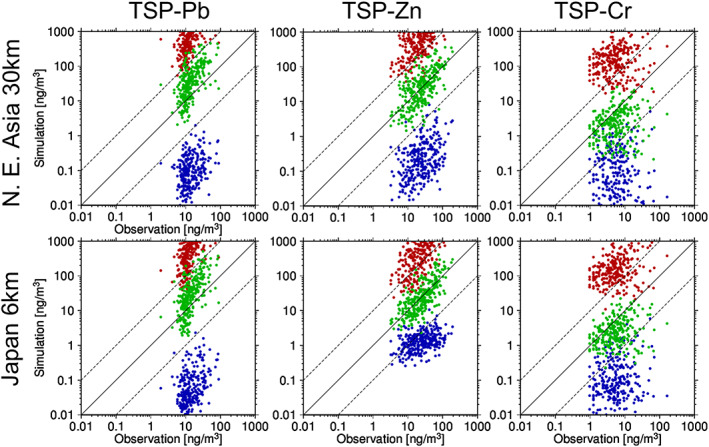
Same as Figure 3 but for Pb, Zn, and Cr.

**Figure A3:**
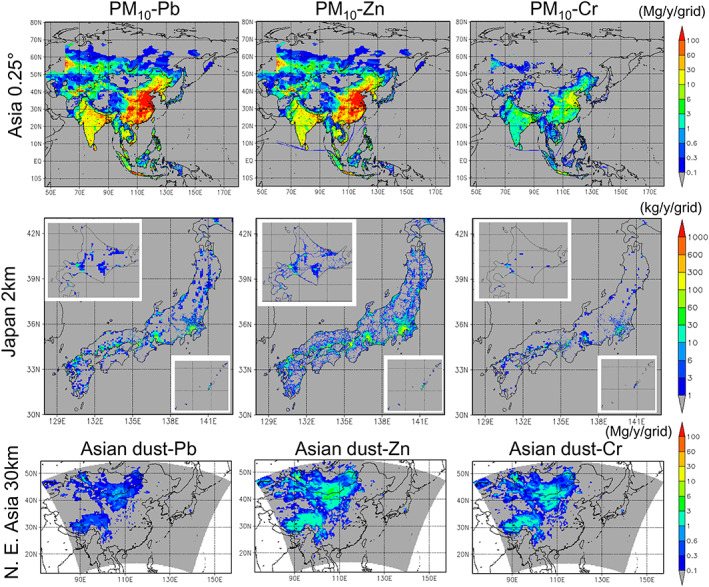
Same as Figure 4 but for Pb, Zn, and Cr.

## Data Availability

The simulated and observed data used in the figures and tables, including the annual total semi‐bottom‐up emission inventory, are available at https://mri-2.mri-jma.go.jp/owncloud/index.php/s/jbe655aDrBXcw0H (last accessed: 10 November 2019). The emission factors used in the study are provided in Supporting Information S1. In terms of raw data sets, REASv2 can be obtained from https://www.nies.go.jp/REAS (last accessed: 19 April 2019), and the MOEJ PM_2.5_ survey data are available at http://www.env.go.jp/air/osen/pm/monitoring.html (last accessed: 15 April 2019).

## Appendix A Pb, Zn, and Cr

**Table A1:** Same as Table 4 but for Pb, Zn, and Cr

	*N* ^a^	Obs. Med.^b^	*Sim:Obs* ^c^	*R* ^d^	*Fa2* ^e^	*Fa5* ^f^
Unit	‐	ng m^−3^	‐	‐	‐	‐
Pb	138 (147)	9.62 (9.47)	4.3 (5.7)	0.48 (0.39)	0.022 (0.0068)	0.66 (0.46)
Zn	134 (143)	29.4 (28.6)	1.3 (1.7)	0.37 (0.31)	0.82 (0.68)	0.98 (0.97)
Cr	135 (144)	1.09 (1.06)	2.3 (1.9)	0.21 (0.40)	0.37 (0.51)	0.88 (0.92)

**Table A2:** Same as Table 5 but for Pb, Zn, and Cr

	*N*	Obs. Med.	*Sim:Obs*	*R*	*Fa2*	*Fa5*
Unit	‐	ng m^−3^	‐	‐	‐	‐
Pb	278	12.2	2.2 (3.0)	0.59 (0.56)	0.40 (0.36)	0.85 (0.82)
Zn	297	24.5	0.85 (1.1)	0.69 (0.65)	0.68 (0.61)	0.97 (0.93)
Cr	261	5.26	0.45 (0.42)	0.20 (0.17)	0.45 (0.43)	0.80 (0.73)

**Table A3:** Same as Table 6 but for Pb, Zn, and Cr

	TMI‐Asia	TMI‐Japan	Mineral dust	Sim:Obs	Sim:Obs
Regions	Total/D01/Japan	Japan	D01	D01	D02
Unit	Gg y^−1^	Gg y^−1^	Gg y^−1^	‐	‐
Pb	760/611/1.77	0.456	1.43	5.7	4.3
Zn	593/465/1.50	0.586	5.96	1.7	1.3
Cr	50.0/36.5/0.107	0.130	3.67	1.9	2.3

This section corresponds to sections 4.1, 4.2, and 4.4 for Pb, Zn, and Cr, respectively. The major findings regarding Pb, Zn, and Cr are similar to those for Cu, Mn, Fe, V, and Ni. Figure A1 compares the simulated Pb, Zn, and Cr in anthropogenic PM_2.5_ over D01 with TMI‐Asia (v1.0) and D02 with TMI‐Japan (v1.0) and those observed at the nationwide MOEJ monitoring stations in Japan. Figure A2 compares the simulated Pb, Zn, and Cr in anthropogenic PM_10_ and Asian dust and the observed PB, Zn, and Cr in TSP measured at Yonago. In both figures, the simulations with the semi‐bottom‐up inventories were settled within the ranges of the maximum and minimum estimates of the simulations. Among the three estimates, the average result matched best with the observation.

The statistical metrics for the comparison of Figures A1 and A2 are summarized in Tables A1 and A2 for the assessments of the spatial distributions and temporal variations, respectively. Fairly good correlations were found for Pb and Zn. The simulated Zn quantitatively agreed with the observed value (*Sim:Obs*: 0.85–1.7; *Fa2*: 0.61–0.82; *Fa5*: 0.93–0.98), while the simulated Pb was significantly overestimated with respect to the nationwide PM_2.5_ measurement (*Sim:Obs*: 4.3–5.7; *Fa2*: 0.0068–0.22; *Fa5*: 0.46–0.66) and moderately overestimated with respect to the Yonago TSP measurement (*Sim:Obs*: 2.2–3.0; *Fa2*: 0.36–0.40; *Fa5*: 0.82–0.85). Although the quantitative agreement was not very low (*Fa2*: 0.37–0.51; *Fa5*: 0.73–0.92), the inventory of Cr needs improvement (low *R*).

Figure A3 and Table A3 show the spatial distributions and areal summations of the emission amounts of Pb, Zn, and Cr, respectively. The anthropogenic Asian total emissions of Pb and Zn amounted to 760 and 593 Gg y^−1^, respectively, which were as large as those of Fe (904 Gg y^−1^). It should be noted that the emission of Pb could be overestimated by fourfold to fivefold. The anthropogenic Asian total emission of Cr was 50.0 Gg y^−1^, which was smaller than that of V (72.3 Gg y^−1^) and Ni (75.9 Gg y^−1^). The estimations of Pb and Zn of TMI‐Asia (v1.0) were much larger than those of TMI‐Japan (v1.0), while the opposite was true for the other metals. The emission amounts of Pb and Zn from Asian dust were much smaller than those from anthropogenic origin (approximately 1% or smaller), while the emission amount of Cr from Asian dust accounted for approximately 10% of anthropogenic Cr.

Figure A4 shows the total amounts of Pb, Zn, and Cr and their emission sectors from different areas of Japan. The major contributing sectors of Pb are “incineration,” “iron‐steel industry,” and “metals industry (other than iron‐steel).” “Incineration” was the most important sector for PM_2.5_‐Zn, while “road brake” and “metals industry” were important for PM_10_‐Zn. The contribution of the “others” sector also contributed to 10–25% of PM_10_‐Zn. Among the other sectors, approximately 90% originated from “road tire.” For Cr, “iron‐steel industry” was the major contributor, followed by “incineration.” For PM_2.5_‐Cr, the contributions of “navigation” over the Seto Inland Sea region (in Chugoku‐Shikoku) and “road brake” were found.
