# Supplementary material for: Modeling Transition Metals in East Asia and Japan and Its Emission Sources
Source: Geohealth. 2020 Sep 14;4(9):e2020GH000259. doi: 10.1029/2020GH000259 (PMC7507570; doi:10.1029/2020GH000259)
Supplement: Supplementary file 1 — Supporting Information S1 [file GH2-4-e2020GH000259-s001.docx]

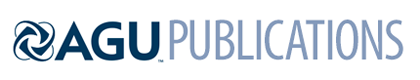


*GeoHealth*

Supporting Information for

**[Modeling transition metals in East Asia and Japan and its emission sources]**

[Mizuo Kajino^1,2^, Hiroyuki Hagino^3^, Yuji Fujitani^4^, Tazuko Morikawa^3^, Tetsuo Fukui^5^, Kazunari Onishi^6^, Tomoaki Okuda^7^, Tomoki Kajikawa^8^, Yasuhito Igarashi^9,10^]

[1Meteorological Research Institute, Japan Meteorological Agency, Japan, ^2^Faculty of Life and Environmental Sciences, University of Tsukuba, Japan, ^3^Japan Automobile Research Institute, Japan, ^4^National Institute for Environmental Studies, Japan, ^5^Institute of Behavioral Sciences, Japan, ^6^St. Luke’s International University, Japan, ^7^Faculty of Science and Technology, Keio University, Japan, ^8^Graduate School of Creative Science and Engineering, Waseda University, Japan, ^9^Institute for Integrated Radiation and Nuclear Science, Kyoto University, Japan, ^10^College of Science, Ibaraki University, Japan ]

**Contents of this file**

Tables S1 to S5.

**Introduction**

Tables S1 and S2 contains emission profiles used for the average estimates of PM_2.5_ and PM_10_ in TMI-Asia v1.0, respectively. Tables S3 and S4 are same as S1 and S2, respectively, but for TMI-Japan v1.0. Table S5 shows emission profile of the Asian mineral dust, obtained from the metal profiles of Certified Reference Material of National Institute for Environmental Studies of Japan (NIES CRM No.30; Gobi Kosa).

Table S1. Emission profiles used for the average estimates of PM_2.5_ TMI-Asia v1.0 (g/g-PM_2.5_ %)

| Sector^a^ | Fe | Cu | Mn | Pb | V | Zn | Co | Ni | Cd | Cr |
| --- | --- | --- | --- | --- | --- | --- | --- | --- | --- | --- |
| (1) | 0.041 | 0.000 | 0.004 | 0.000 | 0.000 | 0.000 | 0.000 | 0.005 | 0.000 | 0.015 |
| (2) | 0.176 | 0.045 | 0.012 | 0.297 | 0.002 | 0.371 | 0.001 | 0.005 | 0.008 | 0.004 |
| (3) | 3.103 | 1.012 | 0.427 | 2.638 | 0.111 | 2.375 | 0.092 | 0.199 | 0.298 | 0.087 |
| (4) | 0.398 | 0.009 | 0.000 | 0.000 | 1.143 | 0.085 | 0.007 | 0.471 | 0.010 | 0.057 |
| (5) | 0.010 | 0.000 | 0.000 | 0.030 | 0.000 | 0.030 | 0.000 | 0.000 | 0.010 | 0.000 |
| (6) | 4.177 | 0.068 | 0.073 | 0.131 | 0.661 | 0.261 | 0.008 | 0.226 | 0.003 | 0.033 |
| (7) | 1.800 | 0.130 | 0.031 | 0.200 | 0.013 | 0.300 | 0.010 | 0.018 | 0.000 | 0.040 |
| (8) | 4.177 | 0.068 | 0.073 | 0.131 | 0.661 | 0.261 | 0.008 | 0.226 | 0.003 | 0.033 |
| (9) | 0.541 | 0.036 | 0.031 | 0.110 | 0.011 | 0.270 | 0.002 | 0.014 | 0.007 | 0.017 |

^a^ (1) aviation, (2) domestic, (3) industry, (4) international navigation, (5) other transport, (6) power plants (non-point sources), (7) power plants (non-point sources in Japan), (8) power plants (large point sources), and (9) road transport.

Table S2. Same as Table S1 but for PM_10_ TMI-Asia v1.0 (g/g-PM_10_ %)

| Sector | Fe | Cu | Mn | Pb | V | Zn | Co | Ni | Cd | Cr |
| --- | --- | --- | --- | --- | --- | --- | --- | --- | --- | --- |
| (1) | 0.186 | 0.018 | 0.007 | 0.002 | 0.000 | 0.035 | 0.000 | 0.002 | 0.000 | 0.000 |
| (2) | 0.039 | 0.006 | 0.252 | 1.004 | 0.001 | 0.109 | 0.099 | 0.269 | 0.083 | 0.338 |
| (3) | 3.438 | 1.238 | 0.597 | 3.669 | 0.090 | 2.992 | 0.032 | 0.147 | 0.377 | 0.089 |
| (4) | 0.398 | 0.009 | 0.000 | 0.000 | 1.143 | 0.085 | 0.007 | 0.471 | 0.010 | 0.057 |
| (5) | 0.010 | 0.000 | 0.000 | 0.030 | 0.000 | 0.030 | 0.000 | 0.000 | 0.010 | 0.000 |
| (6) | 4.199 | 0.046 | 0.051 | 0.153 | 0.994 | 0.202 | 0.011 | 0.387 | 0.003 | 0.076 |
| (7) | 1.800 | 0.130 | 0.031 | 0.200 | 0.013 | 0.300 | 0.010 | 0.018 | 0.000 | 0.040 |
| (8) | 4.199 | 0.046 | 0.051 | 0.153 | 0.994 | 0.202 | 0.011 | 0.387 | 0.003 | 0.076 |
| (9) | 0.612 | 0.056 | 0.037 | 0.214 | 0.010 | 0.187 | 0.001 | 0.006 | 0.028 | 0.006 |

Table S3 Emission profiles used for the average estimates of PM_2.5_ TMI-Japan v1.0 (g/g-PM_2.5_ %)

| Sector^a^ | Fe | Cu | Mn | Pb | V | Zn | Co | Ni | Cd | Cr |
| --- | --- | --- | --- | --- | --- | --- | --- | --- | --- | --- |
| (1) | 4.177 | 0.068 | 0.073 | 0.131 | 0.661 | 0.261 | 0.008 | 0.226 | 0.003 | 0.033 |
| (2) | 1.538 | 0.413 | 0.053 | 0.025 | 0.058 | 0.713 | 0.786 | 0.285 | 0.025 | 0.150 |
| (3) | 2.028 | 0.041 | 0.090 | 0.160 | 0.183 | 0.414 | 0.011 | 0.359 | 0.002 | 0.016 |
| (4) | 1.207 | 0.041 | 0.112 | 0.250 | 0.276 | 0.603 | 0.016 | 0.583 | 0.002 | 0.016 |
| (5) | 1.216 | 0.025 | 0.050 | 0.123 | 0.321 | 0.225 | 0.100 | 0.704 | 0.004 | 0.038 |
| (6) | 1.093 | 0.024 | 0.023 | 0.105 | 0.283 | 0.333 | 0.015 | 0.567 | 0.047 | 0.023 |
| (7) | 2.336 | 0.595 | 0.027 | 0.063 | 0.375 | 0.854 | 0.292 | 0.642 | 0.034 | 0.047 |
| (8) | 1.378 | 0.021 | 0.058 | 0.117 | 0.167 | 0.233 | 0.011 | 0.337 | 0.004 | 0.029 |
| (9) | 7.504 | 0.164 | 1.373 | 0.619 | 0.335 | 0.402 | 0.014 | 0.750 | 0.032 | 0.383 |
| (10) | 1.470 | 2.371 | 0.038 | 8.786 | 0.153 | 6.663 | 0.009 | 0.361 | 1.167 | 0.018 |
| (11) | 2.877 | 0.028 | 0.376 | 0.159 | 0.427 | 0.257 | 0.021 | 0.904 | 0.002 | 0.017 |
| (12) | 1.599 | 0.030 | 0.029 | 0.168 | 0.453 | 0.272 | 0.023 | 0.957 | 0.002 | 0.018 |
| (13) | 0.064 | 0.001 | 0.011 | 0.000 | 0.009 | 0.022 | 0.000 | 0.045 | 0.000 | 0.027 |
| (14) | 0.010 | 0.000 | 0.000 | 0.030 | 0.000 | 0.030 | 0.000 | 0.000 | 0.010 | 0.000 |
| (15) | 0.041 | 0.000 | 0.004 | 0.000 | 0.000 | 0.000 | 0.000 | 0.005 | 0.000 | 0.015 |
| (16) | 2.486 | 0.321 | 0.097 | 4.117 | 0.056 | 4.755 | 0.005 | 0.032 | 0.386 | 0.238 |
| (17) | 0.000 | 0.000 | 0.000 | 0.059 | 0.000 | 0.013 | 0.000 | 0.000 | 0.000 | 0.000 |
| (18) | 0.049 | 0.006 | 0.007 | 0.032 | 0.003 | 0.042 | 0.000 | 0.003 | 0.011 | 0.005 |
| (19) | 0.000 | 0.008 | 0.000 | 0.000 | 0.000 | 0.006 | 0.000 | 0.000 | 0.000 | 0.000 |
| (20) | 0.255 | 0.029 | 0.017 | 0.021 | 0.001 | 0.038 | 0.001 | 0.005 | 0.001 | 0.007 |
| (21) | 0.568 | 0.058 | 0.043 | 0.145 | 0.028 | 0.243 | 0.004 | 0.031 | 0.012 | 0.021 |
| (22) | 0.451 | 0.035 | 0.025 | 0.043 | 0.019 | 0.203 | 0.003 | 0.020 | 0.011 | 0.014 |
| (23) | 0.568 | 0.058 | 0.043 | 0.145 | 0.028 | 0.243 | 0.004 | 0.031 | 0.012 | 0.021 |
| (24) | 0.480 | 0.036 | 0.027 | 0.046 | 0.020 | 0.204 | 0.003 | 0.022 | 0.012 | 0.015 |
| (25) | 0.639 | 0.055 | 0.038 | 0.060 | 0.033 | 0.253 | 0.004 | 0.036 | 0.014 | 0.025 |
| (26) | 0.000 | 0.000 | 0.000 | 0.000 | 0.000 | 0.043 | 0.000 | 0.000 | 0.000 | 0.000 |
| (27) | 11.500 | 0.037 | 0.170 | 0.005 | 0.066 | 0.027 | 0.000 | 0.066 | 0.000 | 0.120 |
| (28) | 0.398 | 0.009 | 0.000 | 0.000 | 1.143 | 0.085 | 0.007 | 0.471 | 0.010 | 0.057 |
| (29) | 50.526 | 0.558 | 1.579 | 0.000 | 0.012 | 0.263 | 0.003 | 0.000 | 0.000 | 0.049 |

^a^ (1) electricity industry, (2) heat supply and gas production, (3) non-manufacturing industry (agriculture, forestry, fishery, mining, and building), (4) manufacture (furniture and fitments), (5) manufacture (pulp, paper, and paper craft), (6) manufacture (chemical engineering), (7) manufacture (petroleum and coal products), (8) manufacture (ceramic, stone, and clay products), (9) manufacture (iron and steel), (10) manufacture (non-ferrous metals and products), (11) manufacture (fabricated metal products), (12) manufacture (others), (13) domestic (house and office), (14) operating machine, (15) aviation, (16) incineration, (17) small burn, (18) field burn, (19) smoking, (20) cooking, (21) traffic exhaust (passenger cars), (22) traffic exhaust (bus), (23) traffic exhaust (light duty trucks), (24) traffic exhaust (special use trucks), and (25) traffic exhaust (motor cycles), (26) tire, (27), brake, (28) navigation, and (29) railway

Table S4 Same as Table S3 but for PM_10_ TMI-Japan v1.0 (g/g-PM_10_ %)

| Sector | Fe | Cu | Mn | Pb | V | Zn | Co | Ni | Cd | Cr |
| --- | --- | --- | --- | --- | --- | --- | --- | --- | --- | --- |
| (1) | 4.199 | 0.046 | 0.051 | 0.153 | 0.994 | 0.202 | 0.011 | 0.387 | 0.003 | 0.076 |
| (2) | 0.550 | 0.050 | 0.050 | 0.050 | 0.000 | 0.550 | 2.000 | 0.550 | 0.050 | 0.550 |
| (3) | 1.479 | 0.047 | 0.144 | 0.350 | 0.369 | 0.718 | 0.009 | 0.643 | 0.002 | 0.020 |
| (4) | 0.939 | 0.040 | 0.133 | 0.294 | 0.301 | 0.603 | 0.007 | 0.526 | 0.002 | 0.015 |
| (5) | 1.288 | 0.032 | 0.073 | 0.219 | 0.548 | 0.189 | 0.174 | 1.005 | 0.007 | 0.062 |
| (6) | 0.950 | 0.034 | 0.029 | 0.146 | 0.353 | 0.140 | 0.008 | 0.612 | 0.027 | 0.016 |
| (7) | 0.993 | 0.021 | 0.023 | 0.138 | 0.541 | 0.111 | 0.008 | 0.823 | 0.002 | 0.020 |
| (8) | 0.824 | 0.019 | 0.046 | 0.182 | 0.272 | 0.127 | 0.008 | 0.429 | 0.009 | 0.029 |
| (9) | 9.051 | 0.201 | 1.754 | 0.840 | 0.377 | 0.377 | 0.005 | 0.644 | 0.040 | 0.375 |
| (10) | 1.315 | 2.739 | 0.039 | 9.760 | 0.149 | 7.544 | 0.003 | 0.302 | 1.222 | 0.019 |
| (11) | 3.171 | 0.028 | 0.551 | 0.214 | 0.548 | 0.143 | 0.008 | 0.959 | 0.003 | 0.016 |
| (12) | 1.223 | 0.030 | 0.030 | 0.234 | 0.598 | 0.156 | 0.008 | 1.046 | 0.003 | 0.018 |
| (13) | 0.328 | 0.025 | 0.007 | 0.111 | 0.001 | 0.221 | 0.000 | 0.002 | 0.000 | 0.001 |
| (14) | 0.000 | 0.000 | 0.000 | 0.000 | 0.000 | 0.000 | 0.000 | 0.000 | 0.000 | 0.000 |
| (15) | 0.186 | 0.018 | 0.007 | 0.002 | 0.000 | 0.035 | 0.000 | 0.002 | 0.000 | 0.000 |
| (16) | 0.098 | 0.012 | 0.308 | 1.273 | 0.002 | 0.170 | 0.120 | 0.326 | 0.112 | 0.420 |
| (17) | 0.000 | 0.000 | 0.000 | 0.059 | 0.000 | 0.013 | 0.000 | 0.000 | 0.000 | 0.000 |
| (18) | 0.046 | 0.010 | 0.015 | 0.076 | 0.008 | 0.066 | 0.000 | 0.006 | 0.027 | 0.010 |
| (19) | 0.000 | 0.008 | 0.000 | 0.000 | 0.000 | 0.006 | 0.000 | 0.000 | 0.000 | 0.000 |
| (20) | 0.134 | 0.006 | 0.002 | 0.004 | 0.000 | 0.015 | 0.000 | 0.002 | 0.004 | 0.000 |
| (21) | 0.737 | 0.078 | 0.028 | 0.088 | 0.016 | 0.203 | 0.002 | 0.008 | 0.040 | 0.009 |
| (22) | 0.741 | 0.079 | 0.025 | 0.093 | 0.016 | 0.199 | 0.002 | 0.008 | 0.041 | 0.009 |
| (23) | 0.737 | 0.078 | 0.028 | 0.088 | 0.016 | 0.203 | 0.002 | 0.008 | 0.040 | 0.009 |
| (24) | 0.750 | 0.080 | 0.025 | 0.095 | 0.016 | 0.201 | 0.002 | 0.008 | 0.041 | 0.009 |
| (25) | 0.784 | 0.084 | 0.026 | 0.080 | 0.017 | 0.208 | 0.002 | 0.008 | 0.044 | 0.009 |
| (26) | 0.000 | 0.000 | 0.000 | 0.000 | 0.000 | 1.018 | 0.000 | 0.000 | 0.000 | 0.000 |
| (27) | 41.003 | 4.281 | 0.155 | 0.000 | 0.043 | 1.415 | 0.000 | 0.000 | 0.000 | 0.000 |
| (28) | 0.398 | 0.009 | 0.000 | 0.000 | 1.143 | 0.085 | 0.007 | 0.471 | 0.010 | 0.057 |
| (29) | 55.000 | 0.650 | 1.950 | 0.000 | 0.007 | 0.290 | 0.003 | 0.000 | 0.000 | 0.055 |

Table S5 Emission profiles used for Asian dust (mg/kg-dust)

| Sector | Fe | Cu | Mn | Pb | V | Zn | Co | Ni | Cd | Cr |
| --- | --- | --- | --- | --- | --- | --- | --- | --- | --- | --- |
| Asian dust | 38400 | 34.1 | 768 | 22.4 | 0 | 93.1 | 13.7 | 29.1 | 0 | 57.4 |
